# Systematic Review and Meta-Analysis: Phenotypic Correlates of the Autism Polygenic Score

**DOI:** 10.1016/j.jaacop.2025.04.001

**Published:** 2025-04-14

**Authors:** Melanie M. de Wit, Morgan J. Morgan, Ilan Libedinsky, Chloe Austerberry, Sander Begeer, Abdel Abdellaoui, Angelica Ronald, Tinca J.C. Polderman

**Affiliations:** a Vrije Universiteit Amsterdam, Amsterdam, the Netherlands; b Amsterdam Public Health, Amsterdam, the Netherlands; c University of Surrey, Guildford, United Kingdom; d University of Cambridge, Cambridge, United Kingdom; e Amsterdam UMC, Amsterdam, the Netherlands

**Keywords:** autism spectrum disorder, autistic traits, genome-wide association study (GWAS), mental health, polygenic index

## Abstract

**Objective:**

Genetic factors play a substantial role in the etiology of autism and its co-occurrence with other conditions and traits. The primary objective of this study was to clarify the associations between the autism polygenic score and autism diagnosis, autistic traits, and related behavioral and neurobiological traits.

**Method:**

Peer-reviewed studies written in English reporting univariate associations were included. PubMed, Web of Science, PsycINFO, and Scopus were systematically searched on November 2, 2022, and January 6, 2023. The quality of included studies was assessed using the QUIPS tool, systematic review with best-evidence synthesis was applied, and meta-analyses were performed if >5 studies were conducted on similar phenotypes.

**Results:**

Of 72 eligible studies (pooled N = 720,087), 61 received high-quality ratings. Meta-analysis of 9 studies revealed strong evidence for an association between the autism polygenic score and autism diagnosis (meta-analytic *r* = 0.158 [95% CI 0.067-0.249]). The systematic review revealed strong evidence for an association with social behavior, depression, and motor skills and weak evidence for physical activity. Associations with other outcomes were inconclusive, and effect sizes were generally small (median *r* = 0.03).

**Conclusion:**

The autism polygenic score is consistently associated with autism diagnosis and a small number of co-occurring traits. Associations with many other traits and conditions are not significant. Due to its inconsistent associations and limited generalizability, it must be emphasized that the autism polygenic score does not have clinical utility and should be applied only for scientific purposes, with improvements needed for a deeper understanding of the polygenic underpinnings of autism.

**Diversity & Inclusion Statement:**

One or more of the authors of this paper self-identifies as a member of one or more historically underrepresented racial and/or ethnic groups in science. One or more of the authors of this paper self-identifies as a member of one or more historically underrepresented sexual and/or gender groups in science. We actively worked to promote sex and gender balance in our author group. While citing references scientifically relevant for this work, we also actively worked to promote sex and gender balance in our reference list. The author list of this paper includes contributors from the location and/or community where the research was conducted who participated in the data collection, design, analysis, and/or interpretation of the work.

**Study registration information:**

The Association Between Polygenic Scores for Autism Spectrum Disorder and Autism Spectrum Disorder and Associated Traits: A Systematic Review and Meta-analysis; https://www.crd.york.ac.uk/PROSPERO/view/CRD42022307993.

## Plain language summary

This systematic review examined how the autism genetic risk score is linked to autism diagnosis and related traits. The findings showed a consistent link between the autism genetic score and autism diagnosis, as well as some related traits like social behavior, depression, and motor skills, though most other associations were small or inconclusive. The results emphasize that while the autism polygenic score (PGS) provides important insights for research, it lacks clinical utility at this time.

Autism spectrum disorder (hereafter referred to as autism, following Bottema-Beutel *et al.*^1^) is a complex neurodevelopmental condition that is characterized by atypical social interaction and communication, repetitive behavior and focused interests, and sensory sensitivities. The prevalence of autism is estimated at 1% to 2% of the worldwide population,^2^ with genetic factors substantially contributing to its etiology. Meta-analyses of twin and family studies indicate that the heritability of autism is approximately 64% to 91%.^3^

Autism shows considerable co-occurrence with other developmental, psychiatric, and somatic conditions and behavioral traits. The incidence of additional psychiatric diagnoses in people with autism is estimated to be about 70%,^4^ and autistic individuals also have an increased liability for a range of physical conditions.^5^ This phenotypic co-occurrence may in part be explained by shared genetic influences between autism, autistic traits, and neurodevelopmental, psychiatric, and physical conditions.^6^^,^^7^

Genome-wide association studies (GWASs) assess the relation between a large number of common genetic variants spread across the human genome and phenotypic outcomes of interest. The latest autism meta-GWAS by Grove *et al.*^8^ identified 5 independent common genetic loci that were significantly associated with autism. The GWAS also revealed a single nucleotide polymorphism–based heritability, which reflects the proportion of phenotypic variance that is explained by additive effects of genomic variants included in the GWAS, of 11.8%. However, many common genetic loci beyond those that are significant genome-wide are involved in the etiology of autism.^8^

Polygenic scores, derived from GWAS summary statistics, capture part of the combined influence of these common genetic influences on an outcome. They reflect the effect of multiple genetic variants with small individual effects in a single value. They are calculated by summing together a person’s common genetic variants, weighted by their effect size. Polygenic scores are one of the tools that can be employed to increase understanding of the genetic basis of complex traits such as autism and overlap with other traits,^9^ among many other popular methods in molecular genetic studies.^10^ In addition to their scientific value, polygenic scores have potential future clinical value as complementary to existing diagnostic tools.^11^ However, at present, most psychiatric polygenic scores are not accurate for clinical application to individuals.^12^, ^13^, ^14^, ^15^ Further, there are bioethical issues relating to the application of polygenic scores that require careful planning.^11^

The latest autism GWAS led to a wealth of studies assessing the relation between the autism polygenic score and autism diagnosis, autistic traits, and a wide range of related traits, yet a comprehensive review of this literature is notably absent. Hence, the primary objective of this study was to clarify the extent to which the current autism polygenic score is associated with autism diagnosis, autistic traits, and related behavioral and neurobiological traits. Additionally, we conducted a meta-analysis of studies examining the association between the polygenic score and autism diagnosis. By systematically reviewing and meta-analyzing a broad range of studies, we aimed to assess the scientific value of the autism polygenic score in understanding the genetic architecture of autism and co-occurring traits and conditions.

## Method

This study was preregistered with PROSPERO under reference number CRD42022307993 and followed Preferred Reporting Items for Systematic reviews and Meta-Analyses (PRISMA) reporting guidelines (Table S1, available online). Meta-analyses as described in the preregistration could be performed only for autism diagnosis due to a limited number of studies performed on specific phenotypes and heterogeneity in the employed methods. Hence, outcome categories beyond autism diagnosis are presented as a systematic review with best-evidence synthesis, which serves as an alternative method to systematically assess evidence when meta-analyses cannot be performed. We further performed a series of non-preregistered analyses, details of which are described under “Secondary Analyses.”

### Study Selection

We included studies assessing the relation between the autism polygenic score and autism diagnostic status, autistic traits, and behavioral and neurobiological traits. A first systematic search of PubMed, Web of Science, PsycINFO, and Scopus was performed on November 2, 2022, and a second search was performed on January 6, 2023. The search terms per search engine are provided in Table S2, available online. Studies were excluded based on the following criteria:

1. The predictor was not an autism polygenic score based on the latest autism GWAS.^8^
2. The polygenic score was not based on genome-wide results, but included only a selection of single nucleotide polymorphisms.
3. The study was a review.
4. No direct, univariate associations between the autism polygenic score and an outcome variable were reported (eg, polygenic transmission tests, interaction effects, genetic correlations, multivariate associations).
5. The study was not peer reviewed.
6. The study was not written in English.

**Figure 1:**
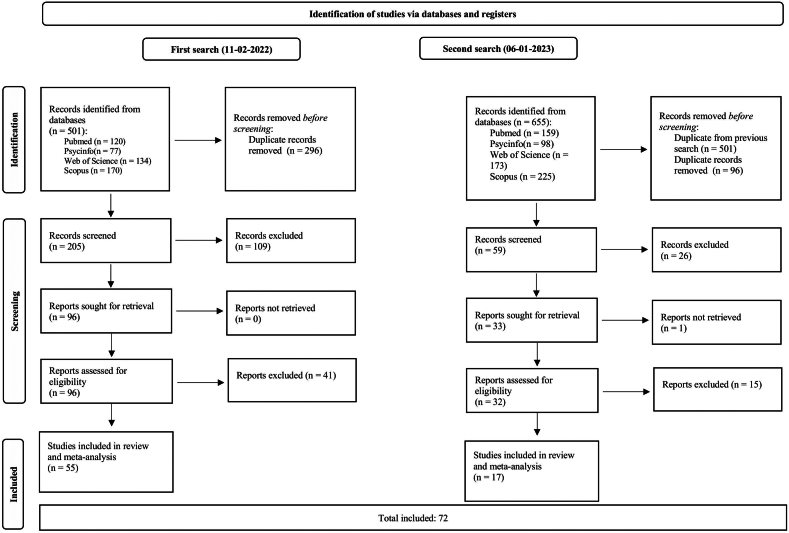
PRISMA Flow Diagram

Inspection of titles and abstracts and full-text screenings were performed by one reviewer (M.M.d.W.). A second reviewer (T.J.C.P.) was consulted in case of any doubt regarding inclusion until consensus was reached. The full selection procedure is shown in Figure 1.

### Quality Assessment

To evaluate the quality, validity, and generalizability of our included studies, we reviewed each study against an adjusted set of quality assessment criteria based on the Quality in Prognostic Studies (QUIPS) tool by Hayden *et al.*^16^^,^^17^ Criteria were largely similar to Ronald *et al.*,^15^ with 1 addition in the control of confounders domain, where we stated that the polygenic score *p* value threshold should be reported. Detailed descriptions of the quality assessment criteria are provided in the Supplementary Methods in Supplement 1, available online. Quality criteria were independently rated by reviewers M.M.d.W. and M.J.M. (rate of agreement 91.8%). As these raters had insufficient specialism in brain-related measures, these specific outcomes were reviewed by an expert (I.L.). In case of any inconsistencies, criteria were assessed independently by a third rater (T.J.C.P.). A quality assessment domain was considered to contain a bias if >50% of the criteria within that domain were scored negatively. A study was considered biased if ≥1 quality assessment domains were biased, and these studies were considered to be of suboptimal quality in the best-evidence synthesis.

### Best-Evidence Synthesis

Meta-analyses were not suitable for outcome categories beyond autism diagnosis due to considerable methodological heterogeneity. Hence, to systematically assess evidence for true associations between the autism polygenic score and our outcome phenotypes in other categories, we applied best-evidence synthesis to our results by assessing the number of studies/statistical tests evaluating an association, the quality of the studies, and the consistency of the findings within these studies. In accordance with previous work,^15^^,^^18^ we defined 4 levels of evidence within a category, as follows:

• Strong evidence: Consistent findings across at least 2 high-quality studies
• Moderate evidence: Consistent findings across 1 high-quality and at least 1 suboptimal quality study
• Weak evidence: Findings of 1 high-quality study or consistent findings in at least 3 or more suboptimal studies
• Inconclusive: Inconsistent findings regardless of study quality or less than 3 suboptimal quality studies available

Findings were considered consistent if 75% or more of the high-quality studies in a category showed statistical significance in the same direction. Best-evidence synthesis was not applied to the 'phenome-wide association study' and 'other' categories.

### Systematic Review and Meta-Analyses

#### Data Extraction

Data were extracted by M.d.W., and data for brain measures were extracted by I.L. If inclusion was uncertain, a second author (T.J.C.P.) was consulted until consensus was reached. When effect sizes were not provided, we contacted the corresponding authors via e-mail. If there was no response but effect sizes were displayed in figures, we extracted the results using an online tool.^19^ All reported effect sizes were transformed to correlation coefficients. An overview of these transformations is presented in the Supplementary Methods in Supplement 1, available online.

Three key criteria guided data extraction. First, the data had to reflect a univariate association between the autism polygenic score and the outcome (covariates were allowed). Second, if studies reported results for multiple polygenic score *p*-value thresholds, we selected the one with the strongest association to improve consistency across studies, as a substantial number of studies reported only the most significant polygenic score *p*-value threshold. Finally, for studies that used multiple models with covariates, we prioritized effect sizes from models with covariates similar to other studies and/or aligned with our quality assessment criteria (sex, age, and socioeconomic status).

#### Primary Analyses

First, we systematically reviewed the significance of individual effect sizes as reported in each study and synthesized these findings narratively in a comprehensive, descriptive table and in a table with extracted effect sizes, all of which provided detailed individual study results.

Second, meta-analyses were performed if a sufficient number of studies (>5) were performed on the same outcome measure (eg, same rater, instrument). This resulted in meta-analytic results for autism diagnostic status only. To account for overlapping samples, we applied 3-level random-effects meta-analyses using the R package metafor.^20^ These models account for dependency between effect sizes by incorporating 3 variance components: sampling variance of extracted effect sizes (level 1), within-sample variance in effect sizes (level 2), and between-sample variance (level 3).^21^ Studies based on the same or largely overlapping samples were grouped as 1 study. We tested for overall significant heterogeneity and heterogeneity within and between studies using the *I*^2^ statistic^22^ and Cochrane *Q*.^23^, ^24^, ^25^ We assessed potential publication bias through funnel plots and Egger tests^26^ and by performing a Rosenthal fail-safe number test at a *p* value of .05.^27^

#### Secondary Analyses

We additionally reported sex differences and a series of secondary meta-analyses, including differences in the association between the autism polygenic score and autism diagnosis for specific populations (Europe-based vs US-based) and meta-regressions to assess the influence of year of publication, sample size, origin of population, sample age (adult >18 years old, children <18 years old, or mixed), and polygenic score calculation method on heterogeneity.^28^

## Results

### Study Characteristics

Studies showed relatively large sample overlap, with 57 unique samples used across 72 studies (pooled N = 720,087). The average percentage of male participants in the included study samples was 48.3% (7 studies^29^, ^30^, ^31^, ^32^, ^33^, ^34^, ^35^ were not included due to missing or unclear sex information). Among the studies, 47.2% were performed on children or adolescents (<18 years old), 19.4% were performed on adults (>18 years old), and 29.2% were performed on mixed samples of children and adults. Age information was unavailable or unclear for 2.8% of the sample.

Study participants lived in Asia (5.6%), Australia (1.4%), Europe (63.8%), and Northern America (25.8%). The remaining studies (6.9%) were based on participants from multiple continents. Genetic ancestry was East Asian (5.6%), European (86.1%), or trans-ancestry (9.7%). Considering that the studies were performed predominantly on participants of European ancestry, results can be considered to reflect participants of European ancestry unless otherwise specified. Individual study characteristics are presented in Tables S5 through S15 in Supplement 2, available online.

### Category Construction

Outcome categories were constructed through consultation and careful consideration between authors M.M.d.W., M.J.M., T.J.C.P., S.B., A.R., and A.A. Categories were loosely based on codes from the *ICD* and the World Health Organization International Classification of Functioning, Disability and Health and resembled the category structure in Ronald *et al.*,^15^ although with some dissimilarities (eg, no addiction category, but a category for emotion recognition). The final set comprised 11 categories based on the identified literature: autism diagnosis, autistic traits, other specific psychiatric classifications, general psychopathology, brain measures, cognition and executive function, emotion recognition, early neurodevelopment, physical well-being, phenome-wide association study, and other. As some outcome categories were rather broad, where possible we described sub-traits within a category. Note that some studies fit into multiple categories.

### Quality Assessment and Levels of Evidence

Of the 72 included studies,^8^^,^^29^, ^30^, ^31^, ^32^, ^33^, ^34^, ^35^, ^36^, ^37^, ^38^, ^39^, ^40^, ^41^, ^42^, ^43^, ^44^, ^45^, ^46^, ^47^, ^48^, ^49^, ^50^, ^51^, ^52^, ^53^, ^54^, ^55^, ^56^, ^57^, ^58^, ^59^, ^60^, ^61^, ^62^, ^63^, ^64^, ^65^, ^66^, ^67^, ^68^, ^69^, ^70^, ^71^, ^72^, ^73^, ^74^, ^75^, ^76^, ^77^, ^78^, ^79^, ^80^, ^81^, ^82^, ^83^, ^84^, ^85^, ^86^, ^87^, ^88^, ^89^, ^90^, ^91^, ^92^, ^93^, ^94^, ^95^, ^96^, ^97^, ^98^, ^99^ 61 did not have any biases, whereas 11 did (9 studies had 1 bias, 1 study had 2 biases, and 1 study had 3 biases). We concluded that the quality of included studies was overall good, and biases were not clustered in specific outcome categories or quality domains. The quality assessments are presented in Table S3 in Supplement 1, available online. The level of evidence, based on best-evidence synthesis, is depicted in Table 1.

**Table 1:** Summary of Results per Outcome Category

Outcome category	No. studies (no. independent cohorts^a^)	No. PGS–phenotype associations tested	No. associations reported as statistically significant	*r* range	Sample size range^b^	Level of evidence for outcome domain	Level of evidence for sub-traits^c^
Autism diagnosis	9 (4)	9	7	0.017 to 0.346	1,238-50,516	Strong	—
Autistic traits	11 (11)	86	10	−0.074 to 0.313	53-13,551	Inconclusive	Strong evidence for social behavior
Specific psychiatric classifications	20 (13)	98	36	−0.106 to 0.213	186-129,169	Inconclusive	Strong evidence for depression
General psychopathology	7 (4)	190	20	−0.143 to 0.292	1,460-10,627	Inconclusive	—
Cognition and executive function	9 (9)	39	5	−0.338 to 0.170	51-212,626	Inconclusive	—
Physical well-being	6 (5)	165	5	−0.114 to 0.095	1,802-16,3619	Inconclusive	Weak evidence for physical activity
Early neurodevelopment	9 (7)	43	7	−0.110 to 0.112	185-19,043	Inconclusive	Strong evidence for motor skills
Emotion recognition	3 (3)	5	3	−0.229 to 0.400	328-4,901	Inconclusive	—
Brain measures	9 (7)	168	10	−0.364 to 0.57	61-32,256	Inconclusive	—
phe-WAS	3 (2)	26,733	15	NA	1,126-33,4976	NA	—
Other	21 (16)	NA	NA	NA	NA	NA	—

Note: NA = not applicable; PGS = polygenic score; phe-WAS = phenome-wide association study; r = correlation.

a The number of independent cohorts describes the use of entirely nonoverlapping samples among studies. However, several study samples contain multiple cohorts and only partly overlap.

b Total sample size range. Range of cases for autism diagnosis category was 134-20,517.

c This column provides information on subdomains for which we did observe an association. A summary of results for domains with inconsistent/negative findings is provided in Tables S5 through S15 in Supplement 2 and Supplementary Results in Supplement 1, available online.

### Systematic Review

The number of studies, independent samples number of polygenic score–phenotype associations tested and associations reported as significant, correlation range, sample size range, and level of evidence per outcome category are depicted in Table 1. Extracted effect sizes are available in Table S4 in Supplement 2, available online. An interactive tool to navigate and explore included studies is provided in Supplement 3, available online.

#### Autism Diagnosis

Nine studies^8^^,^^29^^,^^31^^,^^34^^,^^42^^,^^50^^,^^55^^,^^85^^,^^99^ performed on 4 independent samples assessed the association between the autism polygenic score and autism diagnosis. A histogram and boxplot of the transformed effect sizes within this category are presented in Figures S1 and S2 in Supplement 1, available online. Of the included studies, 7 reported a significant positive association^8^^,^^29^^,^^31^^,^^34^^,^^42^^,^^50^^,^^85^ (5 of which were rated to be high-quality). Of note, 5 of the studies that reported significant associations were performed in the same or largely overlapping sample as the GWAS from which the polygenic score was calculated. Two high-quality studies, based on independent, smaller samples (cases range = 134-295) than the studies that reported significant associations (cases range = 658-20,517), reported no significant association.^55^^,^^99^ In addition, the autism polygenic score was found to distinguish autistic people with intellectual disability from those without intellectual disability.^31^

#### Meta-Analysis on Autism Diagnosis

**Figure 2:**
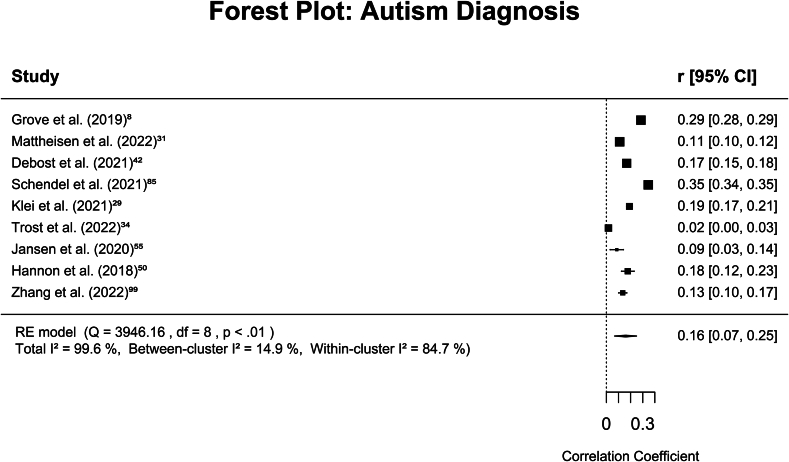
Forest Plot of Meta-Analytic Results for Autism Diagnosis ***Note:*** df *= degrees of freedom;* I*^2^**= test of heterogeneity;* Q *= Cochran* Q*;* r *= (transformed) correlation coefficient*.

Standardized correlation coefficients ranged from 0.017 to 0.346. The overall meta-analyzed effect size was *r* = 0.158 (95% CI 0.067-0.249), indicating a small positive association between the autism polygenic score and autism diagnosis. Total *I*^*2*^ was 99.6%, with between-study value of 14.9% and within-study value of 84.7%. Rosenthal’s fail-safe number was 15,963. An Egger test for funnel asymmetry did not reveal significant asymmetry (*z* = −0.944, *p* = .345) (Figure S3 in Supplement 1, available online). Additional analyses to identify potential sources of heterogeneity did not reveal any significant moderating effects or group differences (Supplementary Results in Supplement 1, available online). The meta-analysis results for the category of autism diagnosis are presented in Figure 2.

#### Autistic Traits

The association between the autism polygenic score and autistic traits was assessed by 11 studies,^35^^,^^36^^,^^39^^,^^68^^,^^72^^,^^82^^,^^87^^,^^90^, ^91^, ^92^, ^93^ in the form of either a total score or specific traits related to repetitive behavior, language and communication, and social behavior. Four studies assessed total scores for autistic traits^36^^,^^72^^,^^90^^,^^91^; 2 (50%) reported significant associations,^90^^,^^91^ of which 1 was performed on a Japanese sample. Studied participants included children^36^^,^^72^^,^^90^^,^^91^ and adults.^36^^,^^72^ In children, the autism polygenic score was associated with overall autistic traits in Japanese^90^ and Swedish^91^ samples, but not in a US sample.^72^ In adults, the autism polygenic score was not associated with overall autistic traits^36^^,^^72^ except that polygenic scores of mothers (but not fathers) of autistic children were associated with their behavior, measured using specific scales of the Broad Autism Phenotype Questionnaire (BAPQ).^72^

With 8 studies,^35^^,^^39^^,^^68^^,^^82^^,^^87^^,^^90^^,^^91^^,^^93^ social behavior was the most commonly studied specific autistic trait. A significant association was reported by 3 (37.5%) of these studies,^82^^,^^87^^,^^90^ one of which was a Japanese sample. Considering the high quality of these studies, this indicates strong evidence for an association with social behavior. Five studies did not find a significant association.^35^^,^^39^^,^^68^^,^^91^^,^^93^ The autism polygenic score was not associated with restricted and repetitive behavior^39^^,^^92^ or with language and communication measures at various ages in childhood^39^^,^^90^^,^^92^ except for language difficulties at age 18 months.^39^ Overall, associations with autistic traits were inconclusive.

### Other Outcome Categories

Due to the magnitude of the available literature, the lack of significant findings, and the inconclusiveness of the evidence according to our best-evidence synthesis, for reasons of conciseness we opted to describe associations with outcomes other than those in autism diagnosis and autistic traits categories only briefly in the main text. More complete narrative descriptions of these categories are presented in the Supplementary Results in Supplement 1, available online; a summary of these results is provided in Table 1; and detailed descriptions per individual study are reported in Tables S5 through S15 in Supplement 2, available online.

#### Specific Psychiatric Classifications

Within this category, several subdomains of psychiatric classifications were identified. We found strong, consistent evidence for an association with depression, whereas no clear pattern could be observed for associations with attention-deficit/hyperactivity disorder (ADHD) diagnosis and traits, suicidal behavior and self-harm, psychosis, eating disorders, and addiction.

#### General Psychopathology

This category included studies that used general mental health questionnaires to assess general psychopathology, either by using the p-factor, which reflects a general propensity toward psychiatric diagnoses, or by analyzing total scores and subscale scores from these questionnaires. No inferences could be drawn due to methodological complexity and mixed findings in terms of significance and direction of effects.

#### Cognition and Executive Function

This category included performance on specific tasks related to these themes, such as working memory, attention performance, inhibitory control, and cognitive flexibility as well as measures of intelligence. Based on mixed findings in terms of significance and direction of effects, we concluded that the evidence for an association with cognition and executive function is inconclusive.

#### Physical Well-Being

This category included phenotypes such as activity levels, general health and health before and during pregnancy, nutrient intake, body mass index, and immune marker levels. We found weak evidence for a possible small association with activity levels, but evidence for associations with nutrient intake, prenatal factors of maternal health and lifestyle, immune marker levels, and body mass index (in people with anorexia) showed no clear observable pattern.

#### Early Neurodevelopment

This category included studies on eye tracking measures and other neurodevelopmental traits such as motor development and temperament in children. We observed consistent evidence for a small association with motor skills and no indications for an association with eye tracking measures.

#### Emotion Recognition

Studies within this category were methodologically diverse, and no clear pattern of associations was observed.

#### Brain Measures

Studies within this category were methodologically diverse, and no clear pattern of associations was observed.

#### Phenome-Wide Association Studies

From 26,733 associations tested in 3 separate studies,^67^^,^^88^^,^^97^ 15 were found to be significant, including factors related to mental and physical health, socioeconomic and demographic characteristics, and emotion recognition.

#### Other

This category included all outcomes not categorized within one of the specific outcome categories and is therefore highly heterogeneous. Most outcomes included here were studied in only 1 article, resulting in limited capacity to draw strong conclusions from the data. The Supplementary Results in Supplement 1, available online, provide more details.

In short, associations between the autism polygenic score and other outcome categories were overall small (median *r* = 0.03) and inconsistent. However, the literature provided strong evidence for an association with social behavior, depression, and motor skills and weak evidence for physical activity.

### Secondary Analyses

Our secondary analyses pointed toward a possible small difference in polygenic score associations between male and female participants for repetitive behavior, communication difficulties, and age at first walking. However, the evidence is limited. Meta-regressions were unable to identify sources of heterogeneity within the autism diagnosis meta-analysis, and no significant differences between US- and European-based samples were observed in the meta-analytic results for autism diagnosis (Figure S4 in Supplement 1, available online).

## Discussion

This study assessed the relation between the autism polygenic score and autism diagnostic status, autistic traits, and behavioral and neurobiological traits. Our results showed strong evidence that the autism polygenic score was associated with autism diagnoses in independent cohorts, which supports the specificity of the polygenic score. We also found strong evidence for an association with a small number of co-occurring phenotypes—social behavior (as an autism characteristic), depression, and motor skills—and weak evidence for an association with physical activity, although results showed incongruency over study samples and should therefore be regarded with caution. The associations with other outcomes were inconclusive, and reported effect sizes were generally small. These results suggest that the autism polygenic score may lack the statistical power to adequately capture polygenic effects. We discuss implications and provide recommendations for future directions.

Although the polygenic score captured some of the established phenotypical associations,^100^^,^^101^ the lack of consistent associations with most co-occurring phenotypes contrasts with extensive literature supporting links between autism and neurodevelopmental, psychiatric, and physical conditions and other traits, as well as genetic associations that have been reported in twin studies and alternative genetic methodologies such as genome-wide restricted maximum likelihood, genome-wide complex trait analysis, and linkage disequilibrium score regression. In contrast to polygenic scores, these methods capture overlap in net genetic effects. Associations with polygenic scores do not necessarily indicate net genetic effects; instead, they represent a correlation between the score and the outcome, which may not capture all underlying genetic influences (explanations of these methods are described in Martin *et al.*^10^).

Our results also highlight disparities between the autism polygenic score and the ADHD polygenic score, with the latter showing stronger (eg, odds ratio between 1.22 and 1.76 for ADHD diagnosis) and more reliable, consistent associations. This is despite comparable high heritability estimates and similar GWAS sample sizes for ADHD and autism, as well as both being a neurodevelopmental condition.^15^ The highly heterogeneous nature of autism could be a potential explanation for this difference.^102^ Increasing evidence suggests that different autism types and characteristics are etiologically distinct, with both rare and common variants influencing its clinical presentation.^103^, ^104^, ^105^ It has been argued that heterogeneity within the autism population could be tempering scientific progress,^102^ and studying autism subtypes^106^ or endophenotypes^107^ would be helpful. The inclusion of heterogeneous samples in the discovery GWAS may have introduced noise and diluted genetic effects. As a consequence, the polygenic scores built on these results may lack the power to identify associations in other populations.

Our results indicate a possible small association between the autism polygenic score and social behavior, a specific domain of autistic traits, but not repetitive behavior and language or communication. Although these results may be partially explained by differences in sample size, it could be speculated that the current autism polygenic score may be unable to capture the full heterogeneous spectrum of autistic characteristics. In line with this idea, early twin studies revealed that the 3 domains of autistic traits, social behavior, communication difficulties, and restricted/repetitive behavior, as described in *DSM-IV,* only modestly correlate genetically, a finding that suggests that largely independent genetic effects may affect different autistic traits.^108^, ^109^, ^110^ As of yet, molecular genetics has not consistently pursued this research direction.^111^ Although some molecular genetic studies were performed on specific domains of autistic traits, large-scale approaches would be needed to explore this direction.^92^^,^^112^^,^^113^ Hence, we propose that GWASs based on individual autistic traits instead of the overarching autism diagnosis and the subsequent polygenic score analyses might enhance our understanding of autism genetics.^114^

Crucially, whereas the autism polygenic score shows some utility in research on a group or population level, current evidence does not support applying it as a prognostic or diagnostic tool for individuals. Moreover, autism is highly complex and heterogeneous, influenced by a wide range of factors beyond common genetic variants, including environmental influences and rare genetic factors. This makes the autism polygenic score unsuitable to ever provide meaningful clinical insights as a stand-alone measure. In the future, depending on statistical improvements to the score, it may offer complementary clinical insights as part of a broad, multidimensional approach.

Limitations of our study include the fact that by focusing on univariate associations and excluding genetic measures and methods beyond polygenic scores based on the most recent, most well-powered GWAS, we may have overlooked important insights into autism genetics. We refer the reader to Akingbuwa *et al.*^115^ for results that are based on methods other than univariate associations with polygenic scores. Second, we note that some studies may have employed partly or completely overlapping samples. This is especially important to note for the autism diagnosis category, as it may have inflated our meta-analytic results. In addition, we highlight that multiple studies included in the meta-analysis were performed on the same or largely overlapping sample as the discovery GWAS, which may have further inflated associations, even when corrections were applied. Furthermore, as some specific phenotypes are not well represented in the available literature, some of our outcome categories included a rather broad selection of phenotypes (eg, specific psychiatric classifications), whereas others were more specific (eg, autism diagnosis). In addition, all categories included studies employing a diversity of methodologies, including differences in instruments and questionnaires used, raters, and polygenic score calculation, which may in part account for the considerable variability in extracted effect sizes and tempers generalizability. Lastly, we point out the considerable heterogeneity in our meta-analysis on the relation between the autism polygenic score and autism diagnosis, which may have caused noise in the estimate. Although we tested potentially moderating variables, we were unable to identify the source of this heterogeneity. A high *I*^2^ value could also be due to low within-sample estimation error, which is likely to occur with large sample sizes such as the ones included in our study.^116^ We advise future studies to identify and reduce such heterogeneity to enhance our understanding of the polygenic nature of autism.

Although we highlight the quality of the included work, we identified some limitations and propose suggestions for future research using the autism polygenic score. A notable observation is the substantial variation in polygenic score calculation methods. Whereas the majority of studies apply methods that select the polygenic score that yields the strongest associations, others used a predefined threshold or employed principal component analyses to construct a polygenic score from multiple thresholds. Similarly, a diverse set of polygenic score calculation programs was applied, including more traditional clumping and thresholding as well as Bayesian approaches. Several recent reports address this issue and advocate for standardized guidelines and reporting practices,^117^^,^^118^ which might improve the comparability of polygenic score findings across studies.

Despite the marked diversity in methods among studies, we observed limited diversity in the participant characteristics. The majority of studies were based on people of European ancestry, resulting in our current understanding of the autism polygenic score being almost solely based on people of European ancestry. This observation is concerning, especially considering the increasing autism prevalence rates in people of color.^117^ Similarly, few included studies addressed understudied subgroups of autism, such as people with intellectual disability or people with autism who are nonverbal. Both scientifically and ethically, it would be of great value to radically improve inclusive research practices.^119^

Despite its limitations, the current autism polygenic score comprises part of a bigger picture and should ideally, as suggested by several recent articles, be integrated with other genetic factors such as common and rare variants, psychiatric family history, and sex in a comprehensive approach to studying autism.^29^^,^^69^^,^^85^^,^^103^ For example, Grove *et al.*^8^ found that a combined polygenic score of autism and correlated traits, such as major depressive disorder and schizophrenia, improved the prediction of autism diagnostic status. Rare genetic variants are not captured by typical polygenic scores, but have been suggested as factors of high influence on autism diagnostic status.^120^ In line with this, Antaki *et al.*^103^ showed that a genetic score containing a combination of common and rare genetic factors significantly increased statistical power over separate common and rare variant scores. In addition, although both psychiatric family history and polygenic scores are supposed to reflect genetic susceptibility, multiple recent studies found that these factors only minimally correlate,^69^^,^^85^^,^^121^^,^^122^ which is likely due to reduced power of the polygenic scores. Schendel *et al.*^85^ therefore argue that they should be considered separate, mostly uncorrelated, measures of the genetic etiology of autism. Lastly, although we did not find significant sex differences in the association of the autism polygenic score with outcome variables, there is growing evidence for a different genetic makeup for men and women with autism. Autistic women on average have a higher number of genetic mutations,^123^ higher mean polygenic load,^88^^,^^103^ and more rare copy number variants load,^124^ albeit a potentially male-biased clinical view on the manifestation of autism might explain diagnostic differences and subsequently observed genetic differences.^125^

In conclusion, we show that the autism polygenic score as a standalone variable is consistently associated with autism diagnostic status but is unable to capture the complex phenotypical and etiological differences of the spectrum and genetic overlap with other traits and conditions. Due to inconsistent associations and limited generalizability, it must be emphasized that the autism polygenic score in its current state does not have clinical utility and should be applied only for scientific purposes. We propose considering the current autism polygenic score as a complementary measure in research, with improvements needed for a more robust understanding of the polygenic underpinnings of autism.

## CRediT authorship contribution statement

**Melanie M. de Wit:** Writing – review & editing, Writing – original draft, Visualization, Project administration, Methodology, Investigation, Formal analysis, Data curation, Conceptualization. **Morgan J. Morgan:** Methodology, Data curation, Writing – review & editing. **Ilan Libedinsky:** Methodology, Data curation, Conceptualization, Writing – review & editing. **Chloe Austerberry:** Writing – review & editing, Methodology, Visualization. **Sander Begeer:** Writing – review & editing, Writing – original draft, Project administration, Conceptualization, Funding acquisition, Methodology. **Abdel Abdellaoui:** Writing – review & editing, Writing – original draft, Visualization, Conceptualization. **Angelica Ronald:** Writing – review & editing, Writing – original draft, Methodology, Conceptualization. **Tinca J.C. Polderman:** Writing – review & editing, Writing – original draft, Methodology, Investigation.
