# Supplementary material for: Systematic Review and Meta-Analysis: Phenotypic Correlates of the Autism Polygenic Score
Source: JAACAP Open. 2025 Apr 14;3(4):839–51. doi: 10.1016/j.jaacop.2025.04.001 (PMC12684455; doi:10.1016/j.jaacop.2025.04.001)
Supplement: Supplement 1 [file mmc1.docx]

**Supplementary materials to ‘Systematic Review and Meta-Analysis: Phenotypic Correlates of the Autism Polygenic Score’**

M.M. de Wit, M.J. Morgan, I. Libedinsky, C. Austerberry, S. Begeer, A. Abdellaoui, A. Ronald and T.J.C. Polderman

**Table of Contents**

[Supplementary Methods 2](#_Toc187149436)

[Quality assessment criteria. 2](#_Toc187149437)

[Effect Size Transformations 4](#_Toc187149438)

[Supplementary Results 4](#_Toc187149439)

[Abbreviations used in the Systematic Review: 4](#_Toc187149440)

[Primary Results 5](#_Toc187149441)

[***Systematic Review*** 5](#_Toc187149442)

[Specific Psychiatric Classifications 5](#_Toc187149443)

[Major depressive disorder 5](#_Toc187149444)

[General psychopathology 7](#_Toc187149445)

[Cognition and Executive Function 8](#_Toc187149446)

[Physical Wellbeing 9](#_Toc187149447)

[Early Neurodevelopment 10](#_Toc187149448)

[Emotion Recognition 10](#_Toc187149449)

[Brain Measures 10](#_Toc187149450)

[Phe-WAS 11](#_Toc187149451)

[Other 11](#_Toc187149452)

[Secondary Results 12](#_Toc187149453)

[***Systematic Review*** 12](#_Toc187149454)

[Sex Differences 12](#_Toc187149455)

[***Meta-Analysis*** 12](#_Toc187149456)

[Secondary Analyses: Meta-Regressions to Identify Sources of Heterogeneity in Meta-analysis 12](#_Toc187149457)

[Secondary Meta-analysis: Population Differences in Polygenic Score Association with Autism Diagnosis 13](#_Toc187149458)

[Supplementary Tables 13](#_Toc187149459)

[Table S1. Search Terms per Search Engine. 13](#_Toc187149460)

[Table S2. PRISMA checklist. 14](#_Toc187149461)

[Table S3. Quality Assessment 20](#_Toc187149462)

[Supplementary Figures Figure S1. Histogram of Effect Sizes Transformed to Correlation Coefficients for Autism Diagnosis 27](#_Toc187149463)

[Figure S2. Boxplot of Effect Sizes Transformed to Correlation Coefficients for Autism Diagnosis 28](#_Toc187149464)

[Figure S3. Funnel Plot of Standard Error for Autism Diagnosis Category 29](#_Toc187149465)

[Figure S4*.* Multi-level Meta-Analysis Results on the Association between Autism Polygenic Score and Autism Diagnosis, including a Test for Population Differences. 30](#_Toc187149466)

[References 30](#_Toc187149467)

# Supplementary Methods

## Quality assessment criteria.

1. Study participation; Study sample adequately represents the population of interest

(A) Description of the key characteristics of the study population (distribution by age, gender and ancestry/ethnicity)

(B) The sampling frame and recruitment are described, including characteristics of the place of recruitment or authors clearly reference where this information can be found

(C) Inclusion and exclusion criteria are described or authors clearly reference where this information can be found

(D) Information about participation at baseline and potential attrition (for genetic data) are described or authors clearly reference where this information can be found

2. Predictor measurement; autism polygenic score is adequately measured

(E) Description of genetic data collection (e.g., blood, saliva) and genotyping (array) is provided, and target sample was not part of GWAS

(F) Genetic data were subject to adequate quality control (minor allele frequency, missing rate, relatedness participants, sex mismatch, and genotype quality), an up-to-date imputation method and an established reference panel was used.

(G) The autism polygenic score is adequately calculated (e.g., pruning/clumping of SNPs), and the p-value threshold for calculating the autism polygenic score is reported.

3. Outcome measurement; Outcome of interest is measured in a similar way for all participants

(H) A clear definition of the outcome measures is provided

(I) Several indications are provided for the validity and reliability of the outcome measure, or a reference is provided.

(J) The method and setting of outcome measurement is the same for all study participants

4. Confounding measurement; Important potential confounders are appropriately accounted for

(K) Age, gender and Socio-Economic Status are accounted for in the analysis

(L) Population stratification and potential batch effects are accounted for in the analysis

(M) In case of clinical samples, treatment and comorbidity are accounted for in the analyses

*5. Analysis and data presentation; Statistical analysis is appropriate*

(N) Sufficient presentation of the data to assess the adequacy of the analytic strategy

(O) The number of participants in the target sample supports sufficient statistical power (N > 400)

(P) The selected statistical model is adequate for the design of the study

(Q) There is no evidence of selective reporting of results, and proper correction for multiple testing was applied.

## **Effect Size Transformations**

To transform effect sizes that were not presented as *b* we applied transformations according to the following formulas. For detailed information regarding effect size calculations and transformations we refer the reader elsewhere^1,2^

Correlation from OR:

b=tanh(​log(OR)/√3​)

Correlation from R2:

r=sign(*b*)×R^2^​

# Supplementary Results

## **Abbreviations used in the Systematic Review:**

ANT: Attention Network Test

BISBAS: Behavioral Inhibition and Behavioral Activation

BRIEF: Behavior Rating Inventory of Executive Function

CBCL: Child Behaviour Checklist

DAWBA: Development and Wellbeing Assessment

GBI: General Behavior Inventory

GSCE: General Certificate of Secondary Education

KSADS: Kiddie Schedule for Affective Disorders and Schizophrenia

NIH: National Institute of Health

OCD: Obsessive Compulsive Disorder

PARCA: Parent Report of Children’s Abilities

SCDC: Social and Communication Disorders Checklist

SDQ: Strengths and Difficulties Questionnaire

UPPS-P: Impulsive Behavior Scale

WASI: Wechsler Abbreviated Scale of Intelligence

WISC: Wechsler Intelligence Scale for Children

WRAT: Wide Range Achievement Test

## **Primary Results**

### ***Systematic Review***

#### Specific Psychiatric Classifications

This category included twenty studies^3–22^assessing the relationship between the autism polygenic score and a broad range of psychiatric classifications or traits: ADHD (n = 2)^3,7^, ADHD traits (n= 2)^4,19^, depression (n = 2)^5,14^, addiction (n = 1)^6103^, suicidal ideation and/or suicide attempt (n = 3)^9,20,21^ eating disorders (n= 2)^11,22^, psychotic spectrum (n= 3)^12,13,16^, nocturnal enuresis (n = 1)^10^ and case-control status for any psychiatric diagnosis (N =1)^8^.

Major depressive disorder*.* The most consistent, strong evidence for an association is for major depressive disorder, for which two studies reported positive associations with lifetime depression (N range = 6847 – 14075)^5,14^ and one reported an association with depression or anxiety symptoms specifically during pregnancy (n = 14,503)^5^.
 *Suicidal behavior and self-harm.* Associations with suicidal behavior and self-harm were inconclusive; In a European cohort, an association with active suicidal ideation was reported^9^, but this was not found in a multi-ethnic cohort. One study reports an association with non-suicidal self-harm but not suicide attempt^20^. When specifically looking at a clinical sample of anorexia patients, no associations with suicidal behavior and self-harm were found^22^.
 *ADHD diagnosis and traits.* For ADHD diagnosis and traits, one study showed a significant association with ADHD diagnosis in a large population sample (N= 11,964 cases and 41,201 controls)^3^, but a significant association was not observed in a clinical sample consisting of 280 cases and 943 controls^7^. Mattheisen et al. found that the autism polygenic score could distinguish people with autism from those with ADHD^3^. In terms of co-occurring diagnoses, the autism polygenic score was found to distinguish people with an ADHD and/or autism diagnosis from controls^3^, whereas in a clinical sample a significant association was not observed^7^. The autism polygenic score was significantly associated with ADHD traits in an east Asian, clinical sample of 1,147 participants with ADHD^23^, but not in two studies in European ancestry samples at various ages^4,19^, except for one specific positive association with inattention and hyperactivity at the age of 8 years (Max N = 7,357)^4^. The latter is interesting, especially considering that the authors applied polygenic score analyses in the same sample of children at 18 months, 36 months, and 5 years (Max N = 11,602), where they did not replicate this result. Based on the mixed evidence in terms of significance and direction both between and within studies, we conclude the evidence for an association with ADHD diagnosis and traits is inconclusive.
 *Psychosis.* Studies on psychotic diagnoses and traits were all performed on clinical samples and findings were inconclusive. The autism polygenic score did not associate with schizophrenia diagnosis in two East Asian samples (N range = 355 - 569)^16,18^, and in an European ancestry sample (N = 1,220 cases), and it positively associated with some, but not all specific domains of the psychotic spectrum (e.g. auditory and visual hallucinations)^13^.
 Eating disorders. No associations were found between the autism polygenic score and disordered eating behavior in two separate studies (N range 3,142 – 3,189)^11,22^, except for increased parent-reported avoidant/restrictive food intake disorder (ARFID).
 *Addiction*. One study reported no significant associations between the autism polygenic score and cannabis use disorder in three different groups consisting of people with schizophrenia, other diagnoses and controls^6^, except for an unexpected negative association that was only present in people diagnosed with DSM classifications other than schizophrenia.
 *Sleep disorders*. No associations were found with insomnia, nocturnal enuresis, and other sleep-related problems^10,15,17^.
 *General psychiatric classifications*. Lastly, one study reported that the autism polygenic score was not associated with the case-control status of any psychiatric classification^56^.
 *Concluding remarks.* The category of specific psychiatric classifications comprised a wide variety of phenotypes, and subsequently, methodological differences. Findings suggest that sample size contributes to the significance of results, but not to the effect size. Participants’ age and ethnicity, and the polygenic score calculation method did not have an influence on the findings. Overall, evidence for an association with psychiatric classifications is inconclusive, except for depression.

#### General psychopathology

Seven studies assessed the association between the autism polygenic score and general psychopathology^24–30^. This category included studies that used general mental health questionnaires to assess general psychopathology, either by using the p-factor, which reflects a general propensity towards psychiatric diagnoses, or by analyzing total scores and subscale scores from these questionnaires.

Assessing overall liability for mental health issues, the autism polygenic score did not consistently associate with the p-factor or total questionnaire scales. Specifically*,* two studies assessed the association between the autism polygenic score and questionnaire total scales, both of which reported a non-significant association with either the CBCL total scale or the KSADS total scale^24,25^.Two studies did not report an association with the p-factor; either constructed from the CBCL^26^ or a p-factor constructed from the DAWBA and SCDC^27^, whereas one did report a significant association with the p-factor constructed from the CBCL^30^
 Questionnaire (Sub)Scales
 For the CBCL, patterns of significant associations with its subscales appear to be random: Gui et al. ^24^ and Thomas et al.^29^ do not report significant associations with any CBCL subscales. Loughnan et al.^25^ only report an association with the caregiver reported inattention subscale of the CBCL, whereas Waszczuk et al. ^30^ applied factor analysis and find several associations with factors composed from the CBCL; externalizing, neurodevelopmental and detachment.

For the KSADS, Loughnan et al.^25^ assessed a total of 48 outcome measures collected from various respondents (caregiver, self-report) and for subsamples of different ancestries (European, non-European, mixed). They report an association with the self-reported depression symptoms in a mixed-ancestry sample and caregiver reported OCD symptoms in a non-European sample, self-reported depression, suicidality symptoms, and caregiver reported ADHD. No associations with other subscales were reported.

For the SDQ, in the ALSPAC sample, Schlag et al.^28^ reported only one association with parent reported low prosociality in 7 year-olds, but not with any other parent or teacher reported low prosociality or peer problems at various ages. In the TEDS sample however, they do find several significant associations (low prosociality at age 7 and 11, peer problems at age 7, 9, 12 and PR peer problems at age 4, 7, 11). Based on these mixed findings in terms of significance and direction of effects, we conclude that the evidence for an association with general psychopathology is inconclusive.

#### Cognition and Executive Function

Nine studies assessed the association between the autism polygenic score and cognition or executive function^23–25,31–36^, which included performance on specific tasks related to these themes such as working memory, attention performance, inhibitory control, cognitive flexibility as well as a measures of intelligence. Only 4 out of 40 measures were reported as significant (10%). Three of these were a positive association with crystallized memory in children in samples of different ancestries (European ancestry N = 5,204, non-European N = 3,964, mixed ancestry N = 9,168, ^25^), a finding that was not replicated in another study performed on an overlapping, European sample of similar size^24^, which may potentially be explained by their different polygenic score calculation methods. Also, Gui et al. report an association with fluid intelligence in one of their two studied population samples, and this could not be replicated in another sample^25^. The other association was a negative association with behavior regulation in 176 children, adolescents and adults that were referred to a specialized hospital unit for autism assessment^35^. The autism polygenic score did not show any associations with executive functioning tasks^23,31,34^, parent-rated executive function beyond behavior regulation^35^, educational attainment at the end of year 11^33^, or cognition at age 4^32^. Based on these mixed findings in terms of significance and direction of effects, we conclude that the evidence for an association with cognition and executive function is inconclusive.

#### Physical Wellbeing

This category comprised six studies^5,14,22,37–39^ on phenotypes such as activity levels, general health and health before and during pregnancy, nutrient intake, BMI and immune marker levels. In terms of activity levels the autism polygenic score was associated with overall reduced activity levels, reduced time spent walking, increased time in sedentary activity and shorter duration of sleep in adults (N = 76,409) measured through accelerometer^37^. We consider this weak evidence for an association with activity levels.
 The autism polygenic score was not associated with nutrient intake in a large (N = 163,619) population-based sample^38^. Two studies, performed on two independent large population-based samples, found no associations of the autism polygenic score with prenatal factors of maternal lifestyle, maternal use of nutritional supplements and medications in pregnancy, maternal illnesses and conditions, biomarkers of nutritional status and toxin exposure in pregnancy and perinatal factors and conditions^5,14^, except for mental health conditions (these are described in more detail in the mental health section). The autism polygenic score was not associated with any of seven tested immune marker levels in a clinical sample of people with schizophrenia or bipolar disorder diagnoses (N = 1,004) except for a decreased level of sIL-2R^39^. Lastly, the autism polygenic score was not associated with minimum BMI during adulthood or minimum BMI during the time of anorexia in patients with a current or previous diagnosis of anorexia nervosa^22^. We conclude that the evidence for an association with physical wellbeing other than activity levels is inconclusive.

#### Early Neurodevelopment

The category of early neurodevelopment included nine studies on eye tracking measures (n = 3) and other neurodevelopmental traits such as motor development and temperament in children^4,36,40–46^. Nine out of 43 (20,9%) tested associations were reported as significant, most of which were related to motor skills; motor difficulties at age 3^4^, age at first walking^42^, overall neuromotor development, overall muscle tone and low and high muscle tone^45^, gross motor skills and receptive language development^46^. Considering we observe significant results in the same direction in multiple high-quality studies, we conclude there is strong evidence for an association with motor skills. Studies using eye tracking assesses association with pupillary light reflex and peak look duration in young children, but no associations were reported^40,41,43^,

#### Emotion Recognition

Three studies^18,47,48^ (N range 345 – 14,901) on the association between the autism polygenic score and emotion recognition found mixed results. Although employing the largest sample within this category, Reed et al. (2020)^47^ do not report a significant association with emotion recognition in healthy participants. Qin et al. (2020)^18^, on the other hand, assessed the association of the autism PGS with emotion recognition in people diagnosed with schizophrenia and controls (N = 345), and find report a negative association of the autism polygenic score with the recognition of negative emotions and total emotion recognition, but not with positive or neutral emotion recognition. Waddington et al. (2021)^48^ studied how the autism polygenic score relates to speed and accuracy of visual and auditory emotion recognition in children diagnosed with ADHD and/or autism (N = 552), and find that it associates with faster visual emotion recognition. In all, evidence for an association with emotion recognition is inconclusive.

#### Brain Measures

Nine studies^18,24,49–55^ assessed the association between the autism polygenic score and brain measures. This category included MRI and EEG measures. The autism polygenic score was significantly positively associated with an accumulated measure of neuroanatomical atypicality for cortical thickness^50^, shorter N290 latency to face vs nonface stimuli^51^, increased salience network connectivity with the postcentral gyrus in autistic and typically developing youth (significant sex differences were observed^53^), functional annotations related to language, executive functions and autism^55^, longer N170 latency to face response^54^, and higher amplitude of low frequency fluctuation in the left amygdala in schizophrenia cases and controls^18^. The autism polygenic score was not associated with cortical measures of autism-related brain regions, including surface area, thickness, and subcortical volume and gyrification measurements^49,55^, nor with resting-state fMRI or diffusion tensor imaging (DTI) measurements^24^, and neither with global and tract-specific fractional anisotropy and mean diffusivity^52^. Based on these mixed findings in terms of significance and direction of effects, we conclude that the evidence for an association with brain measures is inconclusive.

#### Phe-WAS

Three studies applied a phenome-wide association approach^55–57^, where a large number of outcomes is tested for its association with the autism polygenic score in a similar way genetic variants are tested for an association with an outcome in GWAS. From the 3,238 tested variables by Sha et al. (2021)^55^, four (0.001%) showed a significant association with the autism polygenic score (hearing difficulty/problem with background noise, Townsend deprivation index at recruitment, Qualifications: College or University degree and Long-standing illness, disability or infirmity). In the same sample of residents of the UK but using a much larger number of outcomes (23,004), Leppert et al. (2020)^56^ find 10 significant associations, most of which were related to physical health (blood measures, body size, lung function), and some related to mental health (nervousness) and socio-demography. Wendt et al. (2020)^57^ applied phe-was in a US based sample and specifically focused on 491 neurodevelopmental outcomes, and found a significant association with recognition of angry faces and nominal associations with other emotion recognition phenotypes.

#### Other

In the 21 studies^5,16,21,22,32,35,36,55,58–69^ with uncategorized outcome measures, the autism polygenic score was negatively associated with having potentially damaging (rare) genetic variants^62^, age at onset of bipolar disorder^60^ and age at onset of schizophrenia (in a Japanese sample^16^), and was positively associated with female sex^55^, non-righthandedness^55^, paternal age^5^, comorbid conditions related to allergies^63^, higher population density in adult life and moving from rural areas to cities^65^, increased deoxyribonucleic acid (DNA) methylation^59^, childhood trauma^21,67^, experiencing physical/emotional abuse and physical assault^68^. No associations were reported with age^61^, age at first parental concern and age of autism diagnosis^36^, polarity at onset of bipolar disorder^60^, gestational age^32^, vocabulary^61^, reading^61^, parental educational level^35^, several outcomes related to eating disorders (e.g. age of diagnosis, ever been in inpatient or outpatient care^70^), use of depression or anxiety medication^5^, maternal age^5^, response to social skills group training^64^, trajectories of social wariness and preference for solitude^66^, adulthood trauma^67^, sexual abuse^68^.

Some studies reported inconsistent or mixed patterns of associations. For example, the autism polygenic score was negatively associated with the odds of childhood infections in people in the third quartile of the polygenic score, but there was no association in the first, second and fourth quartile^58^. The autism polygenic score was positively associated with hoarding symptoms in some, but not all samples included in Strom et al. (2022)^69^, and the meta-analyzed result was only significant when heterogeneity between samples was not accounted for.

## **Secondary Results**

### ***Systematic Review***

#### Sex Differences

Sex differences were assessed in 14 of the 72 included studies. Overall, there is little evidence for sex differences in associations of autism polygenic score with outcomes^19,31,44,45,55,65,65^. Yet some differences were reported; the autism polygenic score had a stronger association in males for repetitive behavior, social communication^4^ and age at first walking^42^. The autism polygenic score had a stronger association in females for childhood trauma^21^, sameness^29^, and psychiatric behavior^24^. Some studies assessed the influences of autism polygenic score in mothers and fathers of autistic children^5,71^, but reported no differences in the associations.

### ***Meta-Analysis***

#### Secondary Analyses: Meta-Regressions to Identify Sources of Heterogeneity in Meta-analysis

We conducted a series of meta-regression analyses to explore potential moderating influences of study-level variables on the transformed effect sizes. None of the assessed variables were significant moderators of the effect size; year of publication (F (1, 7) = 0.988, *p* = 0.353), sample size (F (1, 7) = 4.258, *p* = 0.600), population (F (2, 6) = 1.203, *p* = .363), sample age (F (2, 6) = 1.975, *p* = 0.219), and PGS calculation method (F (3, 5) = 1.610, *p* = 0.299).
 In addition, we applied a leave-one-out framework to test if any of the included studies significantly influenced heterogeneity. However, *I*^2^ remained significant after exclusion of individual studies (*I*^2^ ≥ 98.97, *p* < .001).

#### Secondary Meta-analysis: Population Differences in Polygenic Score Association with Autism Diagnosis

Based on recent work that pointed out how polygenic score accuracy may vary not only between genetic ancestries, but within ancestries too^72^, we performed secondary analyses assessing whether the polygenic score association differed between Europe and US-based samples. For EU-based samples, the association between autism polygenic score and autism diagnosis was *r* = .20 (95% CI 0.12 – 0.28) whereas the association for US-based samples was *r* = .12 (95% 0.02 – 0.21). A test for subgroup differences revealed no significant difference between these populations, *Q* = 2.77 df = 2, *p* = 0.25. Meta-analysis results for autism diagnosis, including subgroup analyses, are presented in Figure 2.

# Supplementary Tables

## Table S1. PRISMA checklist.

| **Section and Topic** | **Item #** | **Checklist item** | **Location where item is reported** |
| --- | --- | --- | --- |
| **TITLE** | | |  |
| Title | 1 | Identify the report as a systematic review. | Title page |
| **ABSTRACT** | | |  |
| Abstract | 2 | See the PRISMA 2020 for Abstracts checklist. | Abstract |
| **INTRODUCTION** | | |  |
| Rationale | 3 | Describe the rationale for the review in the context of existing knowledge. | Main manuscript, introduction |
| Objectives | 4 | Provide an explicit statement of the objective(s) or question(s) the review addresses. | Main manuscript, introduction |
| **METHODS** | | |  |
| Eligibility criteria | 5 | Specify the inclusion and exclusion criteria for the review and how studies were grouped for the syntheses. | Main manuscript, Methods, study selection |
| Information sources | 6 | Specify all databases, registers, websites, organisations, reference lists and other sources searched or consulted to identify studies. Specify the date when each source was last searched or consulted. | Main manuscript, Methods, study selection |
| Search strategy | 7 | Present the full search strategies for all databases, registers and websites, including any filters and limits used. | Supplement 1 material, Table S2 |
| Selection process | 8 | Specify the methods used to decide whether a study met the inclusion criteria of the review, including how many reviewers screened each record and each report retrieved, whether they worked independently, and if applicable, details of automation tools used in the process. | Main manuscript, Methods, Study Selection |
| Data collection process | 9 | Specify the methods used to collect data from reports, including how many reviewers collected data from each report, whether they worked independently, any processes for obtaining or confirming data from study investigators, and if applicable, details of automation tools used in the process. | Main manuscript, Methods, Data extraction |
| Data items | 10a | List and define all outcomes for which data were sought. Specify whether all results that were compatible with each outcome domain in each study were sought (e.g. for all measures, time points, analyses), and if not, the methods used to decide which results to collect. | Main manuscript, Methods, Data extraction |
|  | 10b | List and define all other variables for which data were sought (e.g. participant and intervention characteristics, funding sources). Describe any assumptions made about any missing or unclear information. | Main manuscript, Methods, Data extraction |
| Study risk of bias assessment | 11 | Specify the methods used to assess risk of bias in the included studies, including details of the tool(s) used, how many reviewers assessed each study and whether they worked independently, and if applicable, details of automation tools used in the process. | Main manuscript, Methods, Quality assessment and Best-evidence synthesis sections |
| Effect measures | 12 | Specify for each outcome the effect measure(s) (e.g. risk ratio, mean difference) used in the synthesis or presentation of results. | Main manuscript, Methods, Data extraction |
| Synthesis methods | 13a | Describe the processes used to decide which studies were eligible for each synthesis (e.g. tabulating the study intervention characteristics and comparing against the planned groups for each synthesis (item #5)). | Main manuscript, Methods, Category construction |
|  | 13b | Describe any methods required to prepare the data for presentation or synthesis, such as handling of missing summary statistics, or data conversions. | Main manuscript, Data extraction and Supplement 1, Supplementary methods |
|  | 13c | Describe any methods used to tabulate or visually display results of individual studies and syntheses. | Main manuscript, Best-evidence synthesis and Primary and Secondary analyses |
|  | 13d | Describe any methods used to synthesize results and provide a rationale for the choice(s). If meta-analysis was performed, describe the model(s), method(s) to identify the presence and extent of statistical heterogeneity, and software package(s) used. | Main manuscript, Best-evidence synthesis and Primary and Secondary analyses |
|  | 13e | Describe any methods used to explore possible causes of heterogeneity among study results (e.g. subgroup analysis, meta-regression). | Main manuscript, Secondary analyses |
|  | 13f | Describe any sensitivity analyses conducted to assess robustness of the synthesized results. | Main manuscript, Secondary analyses |
| Reporting bias assessment | 14 | Describe any methods used to assess risk of bias due to missing results in a synthesis (arising from reporting biases). | NA |
| Certainty assessment | 15 | Describe any methods used to assess certainty (or confidence) in the body of evidence for an outcome. | Main manuscript, Best-evidence synthesis |
| **RESULTS** | | |  |
| Study selection | 16a | Describe the results of the search and selection process, from the number of records identified in the search to the number of studies included in the review, ideally using a flow diagram. | Figure 1 |
|  | 16b | Cite studies that might appear to meet the inclusion criteria, but which were excluded, and explain why they were excluded. | NA |
| Study characteristics | 17 | Cite each included study and present its characteristics. | Tables S5-S15 |
| Risk of bias in studies | 18 | Present assessments of risk of bias for each included study. | Table S3 |
| Results of individual studies | 19 | For all outcomes, present, for each study: (a) summary statistics for each group (where appropriate) and (b) an effect estimate and its precision (e.g. confidence/credible interval), ideally using structured tables or plots. | Table 1 and Supplementary Tables S4-S15 |
| Results of syntheses | 20a | For each synthesis, briefly summarise the characteristics and risk of bias among contributing studies. | Table 1, Table S3, and section on Quality Assessment |
|  | 20b | Present results of all statistical syntheses conducted. If meta-analysis was done, present for each the summary estimate and its precision (e.g. confidence/credible interval) and measures of statistical heterogeneity. If comparing groups, describe the direction of the effect. | Figure 2 |
|  | 20c | Present results of all investigations of possible causes of heterogeneity among study results. | Supplement 1, Supplementary results |
|  | 20d | Present results of all sensitivity analyses conducted to assess the robustness of the synthesized results. | Supplement 1, Supplementary results |
| Reporting biases | 21 | Present assessments of risk of bias due to missing results (arising from reporting biases) for each synthesis assessed. | Figures S1 – S3 |
| Certainty of evidence | 22 | Present assessments of certainty (or confidence) in the body of evidence for each outcome assessed. | Table 1 |
| **DISCUSSION** | | |  |
| Discussion | 23a | Provide a general interpretation of the results in the context of other evidence. | Main manuscript, Discussion |
|  | 23b | Discuss any limitations of the evidence included in the review. | Main manuscript, Discussion |
|  | 23c | Discuss any limitations of the review processes used. | Main manuscript, Discussion |
|  | 23d | Discuss implications of the results for practice, policy, and future research. | Main manuscript, Discussion |
| **OTHER INFORMATION** | | |  |
| Registration and protocol | 24a | Provide registration information for the review, including register name and registration number, or state that the review was not registered. | Main manuscript, Method |
|  | 24b | Indicate where the review protocol can be accessed, or state that a protocol was not prepared. | Main manuscript, Method |
|  | 24c | Describe and explain any amendments to information provided at registration or in the protocol. | Main manuscript, Method |
| Support | 25 | Describe sources of financial or non-financial support for the review, and the role of the funders or sponsors in the review. | Title page |
| Competing interests | 26 | Declare any competing interests of review authors. | Title page |
| Availability of data, code and other materials | 27 | Report which of the following are publicly available and where they can be found: template data collection forms; data extracted from included studies; data used for all analyses; analytic code; any other materials used in the review. | Main manuscript Results Systematic review |

## Table S2. Search Terms per Search Engine.

| PsychInfo  &  Medline | (AB asd OR AB autism OR AB autistic) AND (AB "polygenic score*" OR AB  "polygenic risk score*" OR AB "genetic risk score*" OR AB "genetic score*") |
| --- | --- |
| PubMed | (("ASD"[Title/Abstract] OR "autism"[Title/Abstract] OR "autistic"[Title/Abstract]) AND (("polygenic score*"[Title/Abstract] OR "polygenic risk score*"[Title/Abstract] OR "genetic risk score*"[Title/Abstract]) OR "genetic score*"[Title/Abstract])) |
| Web of  Science | TS=(asd OR autism OR autistic) AND TS=("polygenic score*" OR  "polygenic risk score*" OR "genetic risk score*" OR "genetic score*") |
| Scopus | TITLE-ABS-KEY ( asd OR autism OR autistic ) AND TITLE-ABS-KEY (  "polygenic score*" OR "polygenic risk score*" OR "genetic risk score*" OR "genetic score*" ) |

## Table S3. Quality Assessment

|  | Participants | | | | Predictor | | | Outcome | | | Analyses | | | Confounding | | | | Number of biases |
| --- | --- | --- | --- | --- | --- | --- | --- | --- | --- | --- | --- | --- | --- | --- | --- | --- | --- | --- |
| Study/Criterium | A | B | C | D | E | F | G | H | I | J | K | L | M | N | O | P | Q |  |
| **Autism diagnosis** | | | | | | | | | | | | | | | | | | |
| Debost et al. (2022) | + | + | + | + | + | - | + | +- | + | - | - | + | +- | +- | + | + | +- | 0 |
| Grove et al. (2019) | + | + | + | + | +- | + | + | + | + | - | - | + | - | + | + | + | + | 0 |
| Hannon et al. (2018) | +- | + | + | + | + | +- | + | + | + | + | +- | + | +- | + | + | + | + | 0 |
| Jansen et al. (2020) | +- | + | + | + | + | + | + | + | + | + | +- | + | +- | + | + | + | + | 0 |
| Klei et al. (2021) | - | - | - | - | +- | + | + | - | - | - | - | - | - | + | + | + | + | 3 |
| Mattheisen et al. (2022) | +- | + | + | + | - | + | + | +- | + | - | - | +- | +- | +- | + | + | +- | 0 |
| Schendel et al. (2022) | + | + | + | + | - | +- | + | + | + | + | +- | + | +- | + | + | + | + | 0 |
| Trost et al. (2022) | +- | - | - | - | + | +- | + | + | + | - | - | - | - | + | + | + | +- | 2 |
| Zhang et al. (2022) | +- | + | + | + | +- | - | + | + | + | +- | +- | + | +- | + | + | + | + | 0 |
| **Autistic traits** | | | | | | | | | | | | | | | | | | |
| Askeland et al. (2022) | + | + | +- | + | + | + | + | + | + | + | +- | + | NA | + | + | + | + | 0 |
| Li et al. (2020) | +- | + | + | + | + | + | + | +- | + | + | +- | + | +- | + | - | + | + | 0 |
| Nayar et al. (2021) | + | + | + | + | +- | + | +- | + | + | + | - | + | NA | + | + | + | + | 0 |
| Reed et al. (2021) | + | + | +- | + | + | + | + | + | + | + | - | + | NA | + | + | + | + | 0 |
| Riglin et al. (2021) | + | + | + | + | +- | + | + | + | + | + | - | - | NA | + | + | + | - | 0 |
| Serdarevic et al. (2020) | + | + | + | + | + | + | + | + | + | + | +- | + | NA | + | + | + | + | 0 |
| Takahashi et al. (2020) | + | + | + | + | +- | + | + | + | + | + | - | + | NA | + | + | + | +- | 0 |
| Taylor et al. (2019) | +- | + | + | + | + | + | + | + | + | +- | +- | + | - | +- | + | + | +- | 0 |
| Thomas et al. (2022) | + | + | + | + | - | + | - | + | + | - | +- | + | - | + | + | + | + | 1 |
| Torske et al. (2020) | + | + | + | + | + | + | + | + | + | + | +- | + | +- | + | - | + | - | 0 |
| Yap et al. (2021) | - | +- | + | + | + | + | +- | - | + | - | +- | + | - | - | + | + | +- | 1 |
| **Specific psychiatric classifications** | | | | | | | | | | | | | | | | | | |
| Askeland et al. (2022) | + | + | +- | + | + | + | + | + | + | + | +- | + | NA | + | + | + | + | 0 |
| Chang et al. (2020) | + | + | + | - | +- | + | + | + | + | + | +- | +- | +- | + | + | + | + | 0 |
| Havdahl et al. (2022) | + | + | - | + | +- | + | + | + | - | + | - | + | NA | + | + | + | + | 0 |
| Hjorthøj et al. (2019) | - | + | + | + | +- | - | + | + | +- | - | +- | - | - | + | + | + | + | 0 |
| Jansen et al. (2020) | +- | + | + | + | + | + | + | + | + | + | +- | + | +- | + | + | + | + | 0 |
| Jansen et al. (2021) | + | + | + | + | + | + | + | + | + | +- | - | + | - | + | + | + | + | 0 |
| Joo et al. (2022) | + | + | + | + | + | + | + | + | + | + | + | + | NA | + | + | + | +- | 0 |
| Jorgenson et al. (2021) | +- | + | + | + | +- | + | + | + | + | - | - | + | +- | + | + | + | +- | 0 |
| Koomar et al. (2021) | + | + | +- | + | - | + | + | +- | + | + | - | + | - | + | + | + | +- | 0 |
| Legge et al. (2019) | + | + | + | + | +- | + | + | + | - | + | - | + | +- | + | + | + | +- | 0 |
| Legge et al. (2021) | + | + | - | - | +- | + | + | + | + | - | +- | + | - | + | + | + | + | 0 |
| Leppert et al. (2019) | +- | + | - | + | +- | +- | + | + | - | + | - | + | NA | + | + | + | + | 0 |
| Mattheisen et al. (2022) | - | + | + | + | - | + | + | +- | + | - | - | + | - | - | + | + | +- | 0 |
| Niarchou et al. (2022) | + | + | + | + | +- | + | +- | + | +- | - | +- | + | - | +- | +- | + | + | 0 |
| Ohi et al. (2020) | + | + | + | + | + | + | +- | +- | + | +- | - | - | - | +- | - | + | + | 1 |
| Ohi et al. (2021) | + | + | - | +- | + | +- | + | + | + | + | - | +- | NA | + | + | + | + | 0 |
| Qin et al. (2020) | +- | + | + | + | + | +- | + | + | + | +- | +- | + | - | +- | - | + | + | 0 |
| Riglin et al. (2021) | + | + | + | + | +- | + | + | + | + | + | - | - | NA | + | + | + | - | 0 |
| Russell et al. (2021) | - | + | - | + | +- | + | + | + | - | + | - | - | NA | + | + | + | +- | 1 |
| Warrier & Baron-Cohen (2021) | + | + | + | + | +- | +- | + | + | + | + | +- | + | +- | + | +- | + | + | 0 |
| Zhang et al. (2022) | +- | + | + | + | +- | - | + | + | + | +- | +- | + | +- | + | + | + | + | 0 |
| **General psychopathology** | | | | | | | | | | | | | | | | | | |
| Y. Gui et al. (2022) | + | + | +- | + | - | + | - | + | +- | + | +- | + | NA | +- | + | +- | + | 1 |
| Loughnan et al. (2022) | + | +- | - | + | + | + | + | +- | + | + | + | + | NA | + | + | + | + | 0 |
| Pat et al. (2022) | + | + | + | + | + | + | + | + | + | + | - | + | NA | + | + | + | + | 0 |
| Riglin et al. (2020) | + | + | + | + | +- | + | + | + | + | + | - | - | NA | + | + | +- | +- | 1 |
| Schlag et al. (2022) | + | +- | + | +- | +- | + | + | + | + | - | +- | + | NA | + | + | + | + | 0 |
| Thomas et al. (2022) | + | + | + | + | - | + | - | + | + | - | +- | + | - | + | + | + | + | 1 |
| Waszczuk et al. (2023) | + | + | + | + | + | + | + | + | + | + | - | + | NA | +- | + | + | + | 0 |
| **Cognition and executive functioning** | | | | | | | | | | | | | | | | | | |
| Aguilar-Lacasaña et al. (2022) | + | + | + | + | + | + | + | + | + | + | +- | + | NA | + | + | + | + | 0 |
| Chang et al. (2020) | + | + | + | - | +- | + | + | + | + | + | +- | +- | +- | + | + | + | + | 0 |
| Cullen et al. (2021) | + | + | + | + | + | + | +- | + | + | + | +- | + | NA | + | + | + | + | 0 |
| Y. Gui et al. (2022) | + | + | +- | + | - | + | - | + | +- | + | +- | + | NA | +- | + | +- | + | 1 |
| Hughes et al. (2021) | + | + | + | + | +- | + | + | + | + | + | +- | + | NA | + | + | + | +- | 0 |
| Loughnan et al. (2022) | + | +- | - | + | + | + | + | +- | + | + | + | + | NA | + | + | + | + | 0 |
| Price et al. (2018) | + | + | + | + | + | + | + | + | + | - | - | + | NA | + | + | + | +- | 0 |
| Torske et al. (2020) | + | + | + | + | + | + | + | + | + | + | +- | + | +- | + | - | + | - | 0 |
| Yap et al. (2021) | - | +- | + | + | + | + | +- | - | + | - | +- | + | - | - | + | + | +- | 1 |
| **Early neurodevelopment** | | | | | | | | | | | | | | | | | | |
| Askeland et al. (2022) | + | + | +- | + | + | + | + | + | + | + | +- | + | NA | + | + | + | + | 0 |
| Fish et al. (2021) | +- | - | - | + | + | + | + | + | + | +- | - | + | - | + | - | + | + | 0 |
| A. Gui et al. (2020) | + | + | + | + | + | + | + | + | + | + | - | +- | NA | + | - | + | +- | 0 |
| Hannigan et al. (2023) | + | + | + | + | + | + | + | + | +- | + | +- | + | NA | + | + | + | + | 0 |
| Portugal et al. (2022) | + | + | + | + | +- | +- | - | + | + | + | +- | + | NA | + | + | + | + | 0 |
| Riglin et al. (2022) | +- | + | + | + | +- | + | + | + | + | + | +- | + | NA | +- | + | + | + | 0 |
| Serdarevic et al. (2020) | + | + | + | + | + | + | + | + | + | + | +- | + | NA | + | + | + | + | 0 |
| Takahashi et al. (2020) | + | + | + | + | +- | + | + | + | + | + | - | + | NA | + | + | + | +- | 0 |
| Yap et al. (2021) | - | +- | + | + | + | + | +- | - | + | - | +- | + | - | - | + | + | +- | 1 |
| **Physical wellbeing** | | | | | | | | | | | | | | | | | | |
| Dennison et al. (2021) | + | + | + | + | +- | + | +- | + | + | + | +- | + | + | + | + | + | + | 0 |
| Havdahl et al. (2022) | + | + | - | + | +- | + | +- | + | - | + | - | + | NA | + | + | + | + | 0 |
| Hunjan et al. (2021) | + | + | + | + | - | +- | + | + | - | + | + | + | NA | + | + | + | + | 0 |
| Leppert et al. (2019) | +- | + | - | + | +- | +- | + | + | - | + | - | + | NA | + | + | + | + | 0 |
| Werner et al. (2022) | + | + | + | +- | + | + | + | + | + | + | +- | + | - | + | +- | + | + | 0 |
| Zhang et al. (2022) | +- | + | + | + | +- | - | + | + | + | +- | +- | + | +- | + | + | + | + | 0 |
| **Emotion recognition** | | | | | | | | | | | | | | | | | | |
| Qin et al. (2020) | +- | + | + | + | + | +- | + | + | + | +- | +- | + | - | +- | - | + | + | 0 |
| Reed et al. (2021) | + | + | +- | + | + | + | + | + | + | + | - | + | NA | + | + | + | + | 0 |
| Waddington et al. (2021) | + | + | + | + | + | + | + | + | + | +- | +- | + | - | + | - | + | + | 0 |
| **Phe-WAS** | | | | | | | | | | | | | | | | | | |
| Leppert et al. (2020) | + | + | + | + | + | +- | + | +- | - | + | +- | + | NA | + | + | + | + | 0 |
| Sha et al. (2021) | + | + | + | +- | - | +- | + | + | + | +- | +- | + | NA | +- | + | + | + | 0 |
| Wendt et al. (2020) | + | + | + | - | - | + | + | - | - | - | +- | + | NA | + | + | + | +- | 1 |
| **Brain measures** | | | | | | | | | | | | | | | | | | |
| Alemany et al. (2021) | + | + | + | + | + | +- | + | + | + | + | +- | - | NA | + | + | + | + | 0 |
| Ecker et al. (2022) | + | + | + | + | + | + | +- | + | +- | + | - | +- | - | +- | + | +- | + | 0 |
| A Gui et al. (2021) | + | +- | +- | + | + | + | + | + | + | + | - | +- | - | + | - | +- | +- | 0 |
| Y. Gui et al. (2022) | + | + | +- | + | - | + | - | + | +- | + | +- | + | NA | +- | + | +- | + | 1 |
| P. R. Jansen et al. (2019) | + | + | + | + | + | + | +- | + | + | + | +- | + | NA | + | + | + | + | 0 |
| Lawrence et al. (2022) | + | +- | + | - | + | + | +- | + | + | +- | +- | + | - | - | - | + | - | 1 |
| Mason et al. (2022) | + | + | + | + | +- | +- | + | + | + | +- | - | - | - | + | - | + | - | 1 |
| Qin et al. (2020) | +- | + | + | + | + | +- | + | + | + | +- | +- | + | - | +- | - | + | + | 0 |
| Sha et al. (2021) | + | + | + | +- | - | +- | + | + | + | +- | +- | + | NA | + | + | + | + | 0 |
| **Other** | | | | | | | | | | | | | | | | | | |
| Cullen et al. (2021) | + | + | + | + | + | + | +- | + | + | + | +- | + | NA | + | + | + | + | 0 |
| Debost et al. (2022) | + | + | + | + | + | - | + | +- | + | - | - | + | +- | +- | + | + | +- | 0 |
| Hannon et al. (2018) | +- | + | + | + | + | +- | + | + | + | + | +- | + | +- | + | + | + | + | 0 |
| Havdahl et al. (2022) | + | + | - | + | +- | + | +- | + | - | + | - | + | NA | + | + | + | + | 0 |
| Kalman et al. (2021) | +- | + | + | - | +- | + | + | + | +- | - | - | + | +- | + | + | + | + | 0 |
| Khundrakpam et al. (2020) | + | + | + | + | + | + | + | + | + | +- | +- | + | NA | +- | - | + | + | 0 |
| Klei et al. (2021) | - | - | - | - | +- | + | + | - | - | - | - | - | - | + | + | + | + | 3 |
| Klein et al. (2022) | - | + | + | + | +- | + | + | - | +- | +- | +- | + | +- | +- | + | + | +- | 0 |
| Li et al. (2020) | +- | + | + | + | + | + | + | +- | + | + | +- | + | +- | + | - | + | + | 0 |
| Maxwell et al. (2021) | + | + | +- | + | + | + | +- | + | + | + | +- | + | NA | + | + | + | + | 0 |
| Morneau‐Vaillancourt et al. (2021) | +- | + | +- | + | + | + | +- | + | +- | - | - | + | NA | + | + | + | + | 0 |
| Ohi et al. (2020) | + | + | + | + | + | + | +- | +- | + | +- | - | - | - | +- | - | + | + | 1 |
| Peel et al. (2022) | + | + | + | + | + | + | + | + | +- | + | +- | + | NA | + | + | + | + | 0 |
| Ratanatharathorn et al. (2021) | + | + | + | + | + | +- | + | + | +- | + | +- | + | NA | + | + | + | +- | 0 |
| Sha et al. (2021) | + | + | + | +- | - | +- | + | + | + | +- | +- | + | NA | +- | + | + | + | 0 |
| Strom et al. (2022) | + | + | +- | + | +- | + | +- | + | + | - | +- | +- | NA | + | + | + | + | 0 |
| Torske et al. (2020) | + | + | + | + | + | + | + | + | + | + | +- | + | +- | + | - | + | - | 0 |
| Warrier & Baron-Cohen (2021) | + | + | + | + | +- | +- | + | + | + | + | +- | + | +- | + | +- | + | + | 0 |
| Yap et al. (2021) | - | +- | + | + | + | + | +- | - | + | - | +- | + | - | - | + | + | +- | 1 |
| Zhang et al. (2022) | +- | + | + | + | +- | - | + | + | + | +- | +- | + | +- | + | + | + | + | 0 |

Note. Studies highlighted in gray have been presented earlier in the table due to them being in multiple outcome categories. A bias is detected when > 50% of the criteria within one domain are scored -. Criterium M was not included when counting biases.

# Supplementary Figures
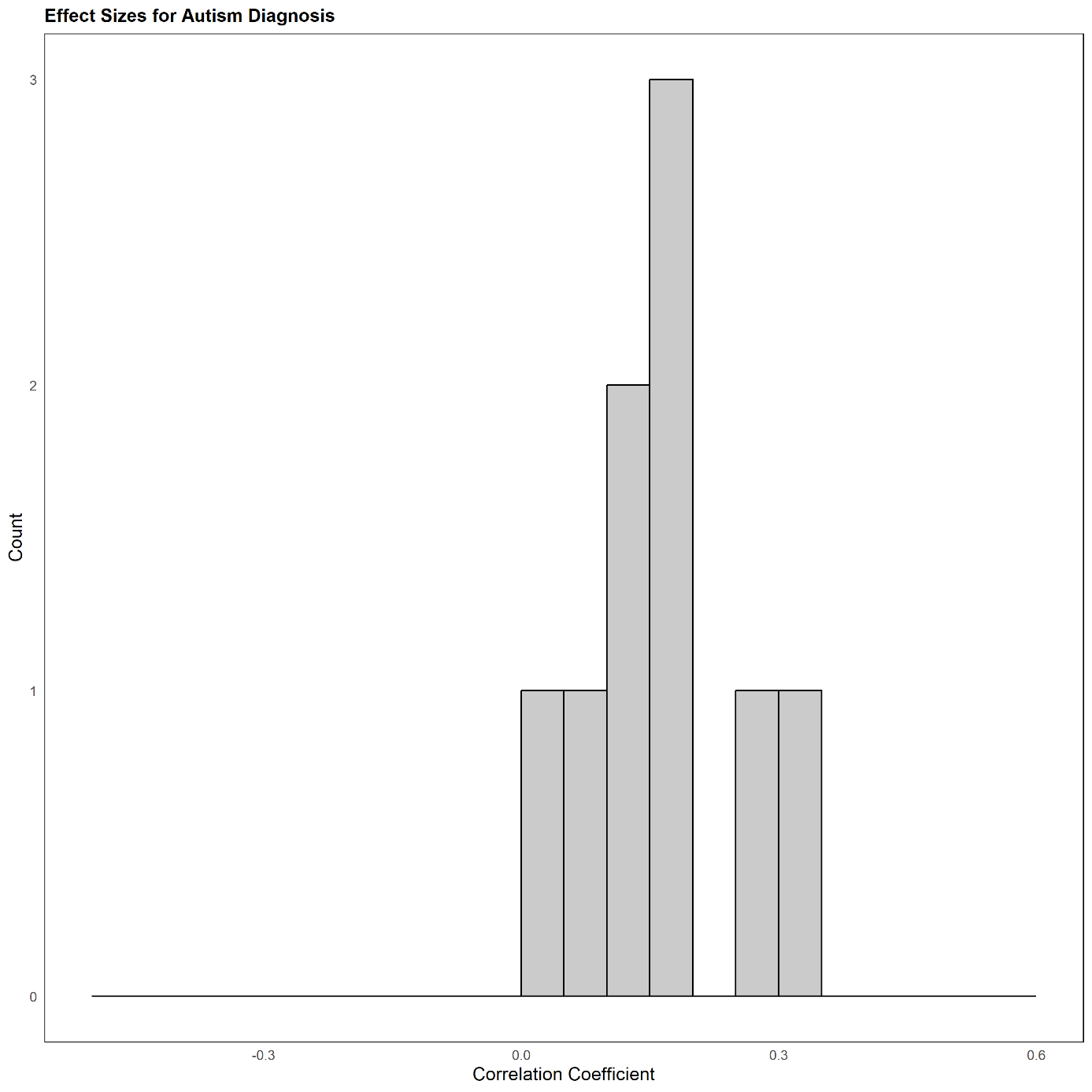
Figure S1. Histogram of Effect Sizes Transformed to Correlation Coefficients for Autism Diagnosis


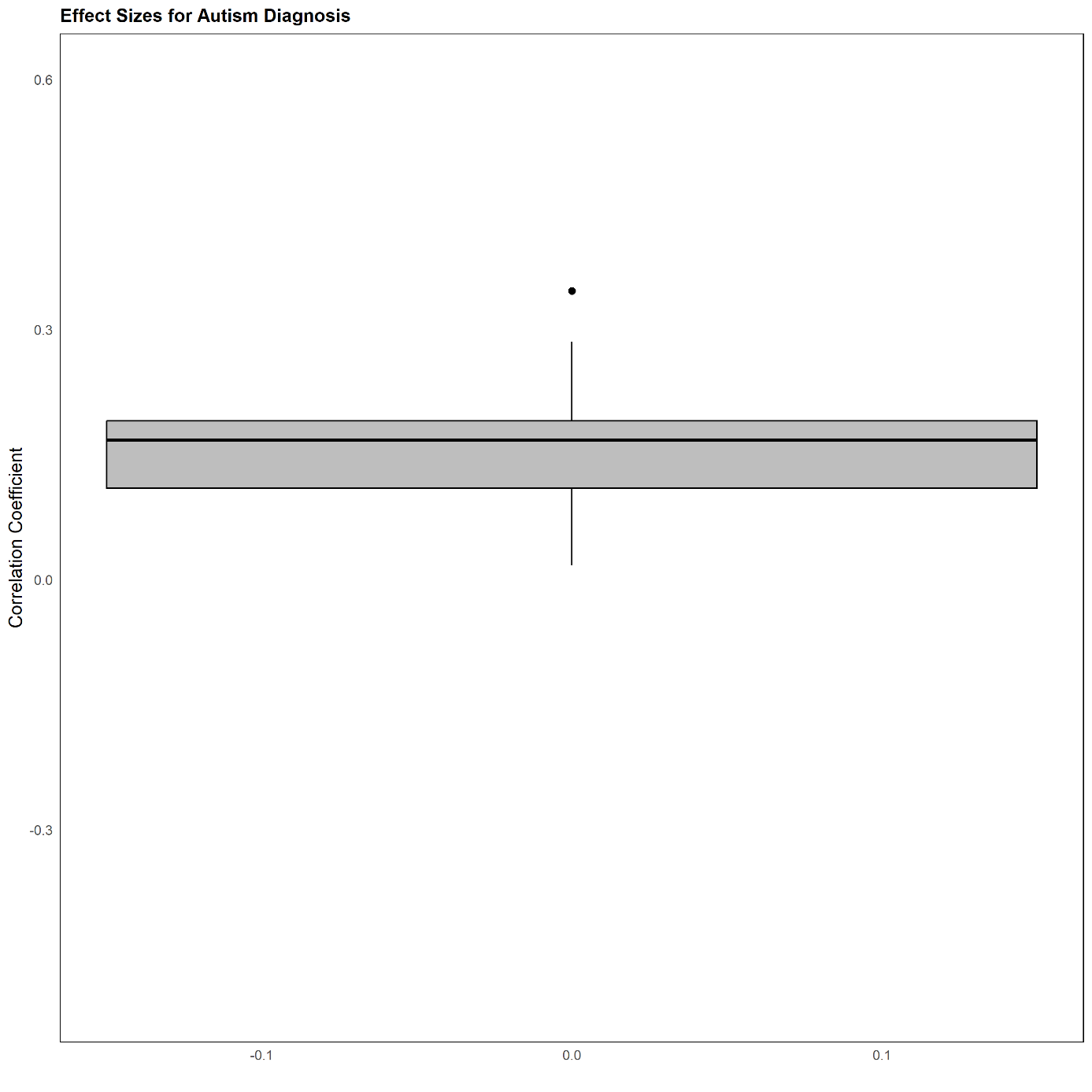


## Figure S2. Boxplot of Effect Sizes Transformed to Correlation Coefficients for Autism Diagnosis


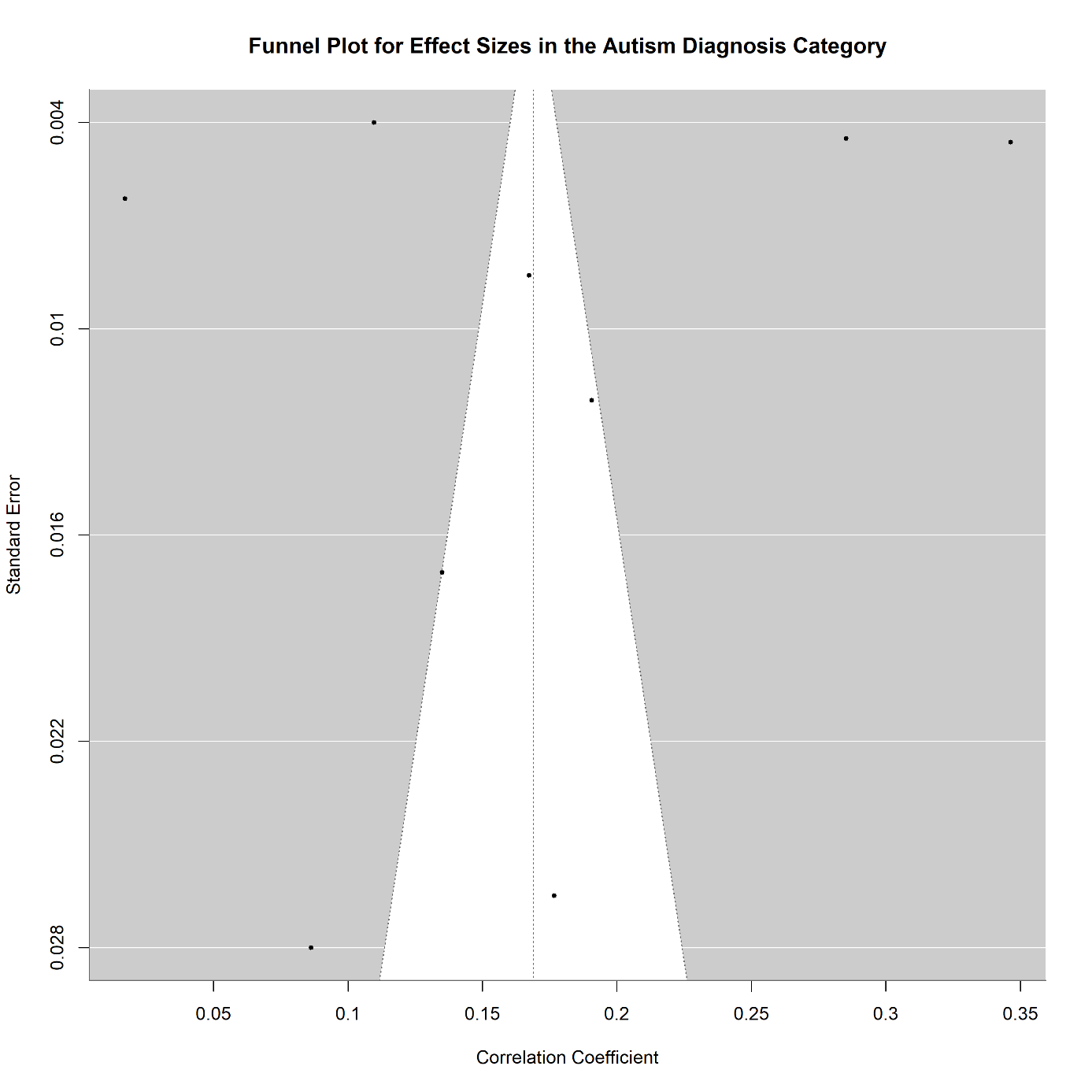

NOTE. Test for Funnel Plot Asymmetry: z = -0.9436, p = 0.3454

Limit Estimate (as sei -> 0): b = 0.2125 (CI: 0.1001, 0.3248)

## Figure S3. Funnel Plot of Standard Error for Autism Diagnosis Category


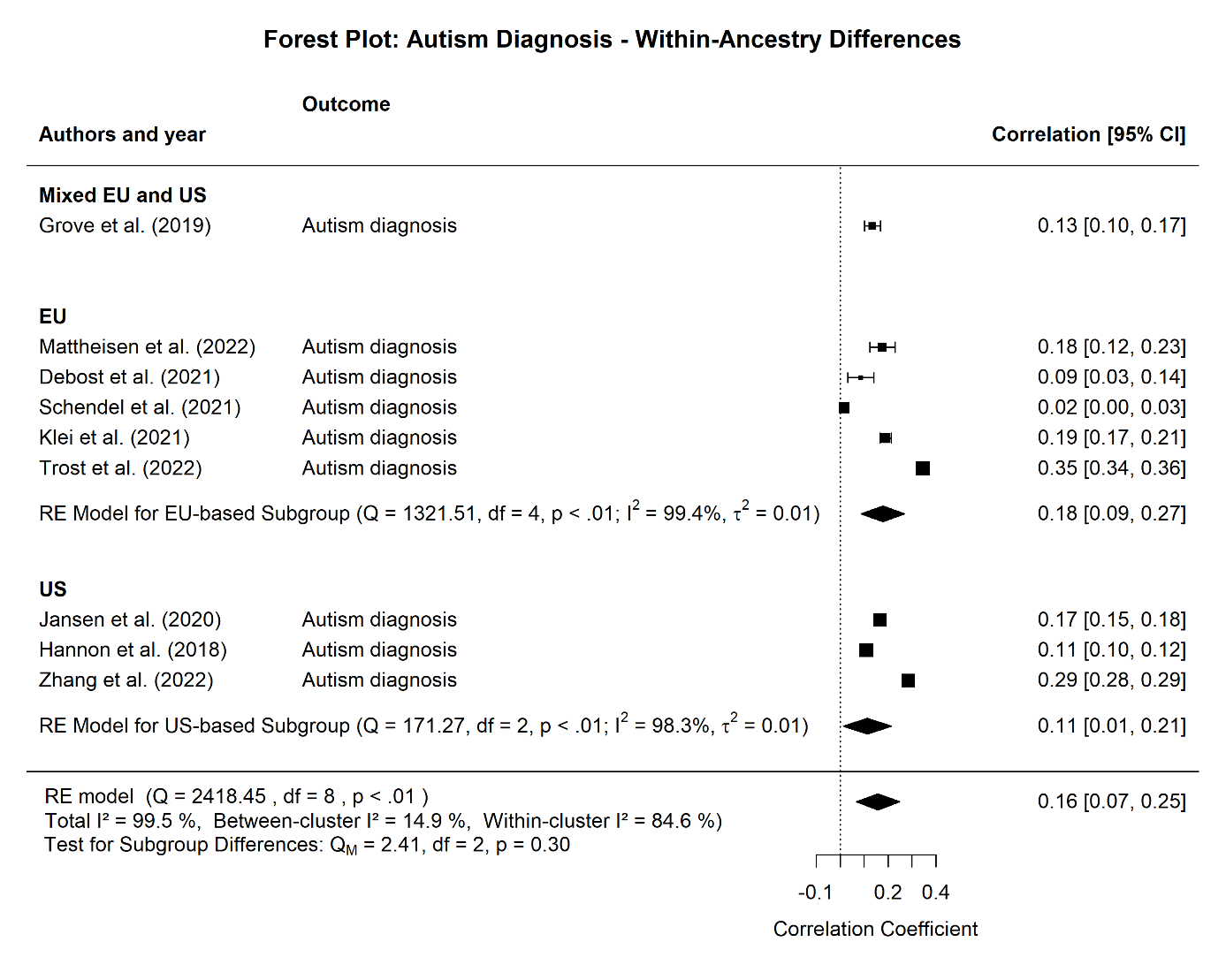


## Figure S4*.* Multi-level Meta-Analysis Results on the Association between Autism Polygenic Score and Autism Diagnosis, including a Test for Population Differences.

# References

1. Borenstein M, Hedges LV, Higgins JPT, Rothstein HR. *Introduction to Meta-Analysis*. John Wiley & Sons; 2021.

2. Cooper HM, Hedges LV, Valentine JC. *The Handbook of Research Synthesis and Meta-Analysis*. 2nd edition. Russell Sage Foundation; 2009.

3. Mattheisen M, Grove J, Als TD, et al. Identification of shared and differentiating genetic architecture for autism spectrum disorder, attention-deficit hyperactivity disorder and case subgroups. *Nat Genet*. 2022;54(10):1470-1478. doi:10.1038/s41588-022-01171-3

4. Askeland RB, Hannigan LJ, Ask H, et al. Early manifestations of genetic risk for neurodevelopmental disorders. *J Child Psychol Psychiatry*. 2021;63(7):810-819. doi:10.1111/JCPP.13528

5. Havdahl A, Wootton RE, Leppert B, et al. Associations Between Pregnancy-Related Predisposing Factors for Offspring Neurodevelopmental Conditions and Parental Genetic Liability to Attention-Deficit/Hyperactivity Disorder, Autism, and Schizophrenia: The Norwegian Mother, Father and Child Cohort Study (MoBa). *JAMA Psychiatry*. 2022;79(8):799. doi:10.1001/jamapsychiatry.2022.1728

6. Hjorthøj C, Uddin MJ, Wimberley T, et al. No evidence of associations between genetic liability for schizophrenia and development of cannabis use disorder. *Psychol Med*. 2021;51(3):479-484. doi:10.1017/S0033291719003362

7. Jansen AG, Dieleman GC, Jansen PR, Verhulst FC, Posthuma D, Polderman TJC. Psychiatric Polygenic Risk Scores as Predictor for Attention Deficit/Hyperactivity Disorder and Autism Spectrum Disorder in a Clinical Child and Adolescent Sample. *Behav Genet*. 2020;50(4):203-212. doi:10.1007/S10519-019-09965-8/FIGURES/1

8. Jansen AG, Jansen PR, Savage JE, et al. The predictive capacity of psychiatric and psychological polygenic risk scores for distinguishing cases in a child and adolescent psychiatric sample from controls. *J Child Psychol Psychiatry*. 2021;62(9):1079-1089. doi:10.1111/JCPP.13370

9. Joo YY, Moon SY, Wang HH, et al. Association of Genome-Wide Polygenic Scores for Multiple Psychiatric and Common Traits in Preadolescent Youths at Risk of Suicide. *JAMA Netw Open*. 2022;5(2):e2148585. doi:10.1001/jamanetworkopen.2021.48585

10. Jørgensen CS, Horsdal HT, Rajagopal VM, et al. Identification of genetic loci associated with nocturnal enuresis: a genome-wide association study. *Lancet Child Adolesc Health*. 2021;5(3):201-209. doi:10.1016/S2352-4642(20)30350-3

11. Koomar T, Thomas TR, Pottschmidt NR, Lutter M, Michaelson JJ. Estimating the Prevalence and Genetic Risk Mechanisms of ARFID in a Large Autism Cohort. *Front Psychiatry*. 2021;12:849. doi:10.3389/FPSYT.2021.668297/BIBTEX

12. Legge SE, Jones HJ, Kendall KM, et al. Association of Genetic Liability to Psychotic Experiences With Neuropsychotic Disorders and Traits. *JAMA Psychiatry*. 2019;76(12):1256-1265. doi:10.1001/JAMAPSYCHIATRY.2019.2508

13. Legge SE, Cardno AG, Allardyce J, et al. Associations Between Schizophrenia Polygenic Liability, Symptom Dimensions, and Cognitive Ability in Schizophrenia. *JAMA Psychiatry*. 2021;78(10):1143-1151. doi:10.1001/JAMAPSYCHIATRY.2021.1961

14. Leppert B, Havdahl A, Riglin L, et al. Association of Maternal Neurodevelopmental Risk Alleles With Early-Life Exposures. *JAMA Psychiatry*. 2019;76(8):834-842. doi:10.1001/JAMAPSYCHIATRY.2019.0774

15. Niarchou M, Singer EV, Straub P, Malow BA, Davis LK. Investigating the genetic pathways of insomnia in Autism Spectrum Disorder. *Res Dev Disabil*. 2022;128:104299. doi:10.1016/j.ridd.2022.104299

16. Ohi K, Nishizawa D, Shimada T, et al. Polygenetic Risk Scores for Major Psychiatric Disorders Among Schizophrenia Patients, Their First-Degree Relatives, and Healthy Participants. *Int J Neuropsychopharmacol*. 2020;23(3):157-164. doi:10.1093/IJNP/PYZ073

17. Ohi K, Ochi R, Noda Y, et al. Polygenic risk scores for major psychiatric and neurodevelopmental disorders contribute to sleep disturbance in childhood: Adolescent Brain Cognitive Development (ABCD) Study. *Transl Psychiatry*. 2021;11(1):187. doi:10.1038/s41398-021-01308-8

18. Qin Y, Kang J, Jiao Z, et al. Polygenic risk for autism spectrum disorder affects left amygdala activity and negative emotion in schizophrenia. *Transl Psychiatry 2020 101*. 2020;10(1):1-12. doi:10.1038/s41398-020-01001-2

19. Riglin L, Leppert B, Langley K, et al. Investigating attention-deficit hyperactivity disorder and autism spectrum disorder traits in the general population: What happens in adult life? *J Child Psychol Psychiatry*. 2021;62(4):449-457. doi:10.1111/JCPP.13297

20. Russell AE, Hemani G, Jones HJ, et al. An exploration of the genetic epidemiology of non-suicidal self-harm and suicide attempt. *BMC Psychiatry*. 2021;21(1):207. doi:10.1186/S12888-021-03216-Z/FIGURES/2

21. Warrier V, Baron-Cohen S. Childhood trauma, life-time self-harm, and suicidal behaviour and ideation are associated with polygenic scores for autism. *Mol Psychiatry*. 2021;26(5):1670-1684. doi:10.1038/s41380-019-0550-x

22. Zhang R, Birgegård A, Fundín B, et al. Association of autism diagnosis and polygenic scores with eating disorder severity. *Eur Eat Disord Rev*. 2022;30(5):442-458. doi:10.1002/erv.2941

23. Chang S, Yang L, Wang Y, Faraone SV. Shared polygenic risk for ADHD, executive dysfunction and other psychiatric disorders. *Transl Psychiatry 2020 101*. 2020;10(1):1-9. doi:10.1038/s41398-020-00872-9

24. Gui Y, Zhou X, Wang Z, et al. Sex-specific genetic association between psychiatric disorders and cognition, behavior and brain imaging in children and adults. *Transl Psychiatry*. 2022;12(1):1-8. doi:10.1038/s41398-022-02041-6

25. Loughnan RJ, Palmer CE, Makowski C, et al. Unique prediction of developmental psychopathology from genetic and familial risk. *J Child Psychol Psychiatry*. 2022;63(12):1631-1643. doi:10.1111/jcpp.13649

26. Pat N, Riglin L, Anney R, et al. Motivation and Cognitive Abilities as Mediators Between Polygenic Scores and Psychopathology in Children. *J Am Acad Child Adolesc Psychiatry*. 2022;61(6):782-795.e3. doi:10.1016/j.jaac.2021.08.019

27. Riglin L, Thapar AK, Leppert B, et al. Using Genetics to Examine a General Liability to Childhood Psychopathology. *Behav Genet*. 2020;50(4):213-220. doi:10.1007/S10519-019-09985-4/TABLES/2

28. Schlag F, Allegrini AG, Buitelaar J, et al. Polygenic risk for mental disorder reveals distinct association profiles across social behaviour in the general population. *Mol Psychiatry*. 2022;27(3):1588-1598. doi:10.1038/s41380-021-01419-0

29. Thomas TR, Koomar T, Casten LG, Tener AJ, Bahl E, Michaelson JJ. Clinical autism subscales have common genetic liabilities that are heritable, pleiotropic, and generalizable to the general population. *Transl Psychiatry*. 2022;12(1):247. doi:10.1038/s41398-022-01982-2

30. Waszczuk MA, Miao J, Docherty AR, et al. General v. specific vulnerabilities: polygenic risk scores and higher-order psychopathology dimensions in the Adolescent Brain Cognitive Development (ABCD) Study. *Psychol Med*. Published online 2021:1-10. doi:10.1017/S0033291721003639

31. Aguilar-Lacasaña S, Vilor-Tejedor N, Jansen PR, et al. Polygenic risk for ADHD and ASD and their relation with cognitive measures in school children. *Psychol Med*. 2022;52(7):1356-1364. doi:10.1017/S0033291720003189

32. Cullen H, Selzam S, Dimitrakopoulou K, Plomin R, Edwards AD. Greater genetic risk for adult psychiatric diseases increases vulnerability to adverse outcome after preterm birth. *Sci Rep 2021 111*. 2021;11(1):1-8. doi:10.1038/s41598-021-90045-5

33. Hughes A, Wade KH, Dickson M, et al. Common health conditions in childhood and adolescence, school absence, and educational attainment: Mendelian randomization study. *Npj Sci Learn 2021 61*. 2021;6(1):1-9. doi:10.1038/s41539-020-00080-6

34. Price KM, Wigg KG, Feng Y, et al. Genome-wide association study of word reading: Overlap with risk genes for neurodevelopmental disorders. *Genes Brain Behav*. 2020;19(6). doi:10.1111/GBB.12648

35. Torske T, Nærland T, Bettella F, et al. Autism spectrum disorder polygenic scores are associated with every day executive function in children admitted for clinical assessment. *Autism Res Off J Int Soc Autism Res*. 2020;13(2):207-220. doi:10.1002/AUR.2207

36. Yap CX, Alvares GA, Henders AK, et al. Analysis of common genetic variation and rare CNVs in the Australian Autism Biobank. *Mol Autism*. 2021;12(1):1-17. doi:10.1186/S13229-020-00407-5/FIGURES/3

37. Dennison CA, Legge SE, Bracher-Smith M, et al. Association of genetic liability for psychiatric disorders with accelerometer-assessed physical activity in the UK Biobank. *PLOS ONE*. 2021;16(3):e0249189. doi:10.1371/JOURNAL.PONE.0249189

38. Hunjan AK, Hübel C, Lin Y, Eley TC, Breen G. Association between polygenic propensity for psychiatric disorders and nutrient intake. *Commun Biol 2021 41*. 2021;4(1):1-9. doi:10.1038/s42003-021-02469-4

39. Werner MCF, Wirgenes KV, Shadrin A, et al. Immune marker levels in severe mental disorders: associations with polygenic risk scores of related mental phenotypes and psoriasis. *Transl Psychiatry*. 2022;12(1):38. doi:10.1038/s41398-022-01811-6

40. Fish LA, Nyström P, Gliga T, et al. Development of the pupillary light reflex from 9 to 24 months: association with common autism spectrum disorder (ASD) genetic liability and 3‐year ASD diagnosis. *J Child Psychol Psychiatry*. 2021;62(11):1308-1319. doi:10.1111/jcpp.13518

41. Gui A, Mason L, Gliga T, et al. Look duration at the face as a developmental endophenotype: elucidating pathways to autism and ADHD. *Dev Psychopathol*. 2020;32(4):1303-1322. doi:10.1017/S0954579420000930

42. Hannigan LJ, Askeland RB, Ask H, et al. Developmental milestones in early childhood and genetic liability to neurodevelopmental disorders. *Psychol Med*. 2023;53(5):1750-1758. doi:10.1017/S0033291721003330

43. Portugal AM, Taylor MJ, Viktorsson C, et al. Pupil size and pupillary light reflex in early infancy: heritability and link to genetic liability to schizophrenia. *J Child Psychol Psychiatry*. 2022;63(9):1068-1077. doi:10.1111/jcpp.13564

44. Riglin L, Tobarra-Sanchez E, Stergiakouli E, et al. Early manifestations of genetic liability for ADHD, autism and schizophrenia at ages 18 and 24 months. *JCPP Adv*. 2022;2(3):e12093. doi:10.1002/jcv2.12093

45. Serdarevic F, Tiemeier H, Jansen PR, et al. Polygenic Risk Scores for Developmental Disorders, Neuromotor Functioning During Infancy, and Autistic Traits in Childhood. *Biol Psychiatry*. 2020;87(2):132-138. doi:10.1016/J.BIOPSYCH.2019.06.006

46. Takahashi N, Harada T, Nishimura T, et al. Association of Genetic Risks With Autism Spectrum Disorder and Early Neurodevelopmental Delays Among Children Without Intellectual Disability. *JAMA Netw Open*. 2020;3(2):1921644. doi:10.1001/jamanetworkopen.2019.21644

47. Reed ZE, Mahedy L, Jackson A, et al. Examining the bidirectional association between emotion recognition and social autistic traits using observational and genetic analyses. *J Child Psychol Psychiatry*. 2021;62(11):1330-1338. doi:10.1111/JCPP.13395

48. Waddington F, Franke B, Hartman C, Buitelaar JK, Rommelse N, Mota NR. A polygenic risk score analysis of ASD and ADHD across emotion recognition subtypes ADHD, ASD, emotion recognition, polygenic risk score, subtyping. *Am J Med Genet*. 2021;186:401-411. doi:10.1002/ajmg.b.32818

49. Alemany S, Blok E, Jansen PR, Muetzel RL, White T. Brain morphology, autistic traits, and polygenic risk for autism: A population-based neuroimaging study. *Autism Res*. 2021;14(10):2085-2099. doi:10.1002/aur.2576

50. Ecker C, Pretzsch CM, Bletsch A, et al. Interindividual Differences in Cortical Thickness and Their Genomic Underpinnings in Autism Spectrum Disorder. *Am J Psychiatry*. 2022;179(3):242-254. doi:10.1176/appi.ajp.2021.20050630

51. Gui A, Meaburn EL, Tye C, Charman T, Johnson MH, Jones EJH. Association of Polygenic Liability for Autism With Face-Sensitive Cortical Responses From Infancy. *JAMA Pediatr*. 2021;175(9):968. doi:10.1001/jamapediatrics.2021.1338

52. Jansen PR, Muetzel RL, Polderman TJC, et al. Polygenic Scores for Neuropsychiatric Traits and White Matter Microstructure in the Pediatric Population. *Biol Psychiatry Cogn Neurosci Neuroimaging*. 2019;4(3):243-250. doi:10.1016/j.bpsc.2018.07.010

53. Lawrence KE, Hernandez LM, Fuster E, et al. Impact of autism genetic risk on brain connectivity: a mechanism for the female protective effect. *Brain*. 2022;145(1):378-387. doi:10.1093/brain/awab204

54. Mason L, Moessnang C, Chatham C, et al. Stratifying the autistic phenotype using electrophysiological indices of social perception. *Sci Transl Med*. 2022;14(658):eabf8987. doi:10.1126/scitranslmed.abf8987

55. Sha Z, Schijven D, Francks C. Patterns of brain asymmetry associated with polygenic risks for autism and schizophrenia implicate language and executive functions but not brain masculinization. *Mol Psychiatry*. 2021;26(12):7652-7660. doi:10.1038/s41380-021-01204-z

56. Leppert B, Millard LAC, Riglin L, et al. A cross-disorder PRS-pheWAS of 5 major psychiatric disorders in UK Biobank. Zhu X, ed. *PLOS Genet*. 2020;16(5):e1008185. doi:10.1371/journal.pgen.1008185

57. Wendt FR, Carvalho CM, Pathak GA, Gelernter J, Polimanti R. Polygenic risk for autism spectrum disorder associates with anger recognition in a neurodevelopment-focused phenome-wide scan of unaffected youths from a population-based cohort. Williams SM, ed. *PLOS Genet*. 2020;16(9):e1009036. doi:10.1371/journal.pgen.1009036

58. Debost JCPG, Thorsteinsson E, Trabjerg B, et al. Genetic and psychosocial influence on the association between early childhood infections and later psychiatric disorders. *Acta Psychiatr Scand*. 2022;146(5):406-419. doi:10.1111/acps.13491

59. Hannon E, Schendel D, Ladd-Acosta C, et al. Elevated polygenic burden for autism is associated with differential DNA methylation at birth. *Genome Med*. 2018;10(1):19. doi:10.1186/s13073-018-0527-4

60. Kalman JL, Loohuis LMO, Vreeker A, et al. Characterisation of age and polarity at onset in bipolar disorder. *Br J Psychiatry*. 2021;219(6):659-669. doi:10.1192/BJP.2021.102

61. Khundrakpam B, Vainik U, Gong J, et al. Neural correlates of polygenic risk score for autism spectrum disorders in general population. *Brain Commun*. 2020;2(2):fcaa092. doi:10.1093/braincomms/fcaa092

62. Klei L, McClain LL, Mahjani B, et al. How rare and common risk variation jointly affect liability for autism spectrum disorder. *Mol Autism*. 2021;12(1):1-13. doi:10.1186/S13229-021-00466-2/FIGURES/5

63. Klein L, D’Urso S, Eapen V, Hwang LD, Lin PI. Exploring polygenic contributors to subgroups of comorbid conditions in autism spectrum disorder. *Sci Rep*. 2022;12(1):3416. doi:10.1038/s41598-022-07399-7

64. Li D, Choque-Olsson N, Jiao H, et al. The influence of common polygenic risk and gene sets on social skills group training response in autism spectrum disorder. *Npj Genomic Med 2020 51*. 2020;5(1):1-8. doi:10.1038/s41525-020-00152-x

65. Maxwell JM, Coleman JRI, Breen G, Vassos E. Association Between Genetic Risk for Psychiatric Disorders and the Probability of Living in Urban Settings. *JAMA Psychiatry*. 2021;78(12):1355. doi:10.1001/jamapsychiatry.2021.2983

66. Morneau‐Vaillancourt G, Andlauer TFM, Ouellet‐Morin I, et al. Polygenic scores differentially predict developmental trajectories of subtypes of social withdrawal in childhood. *J Child Psychol Psychiatry*. 2021;62(11):1320-1329. doi:10.1111/jcpp.13459

67. Peel AJ, Purves KL, Baldwin JR, et al. Genetic and early environmental predictors of adulthood self-reports of trauma. *Br J Psychiatry*. 2022;221(4):613-620. doi:10.1192/bjp.2021.207

68. Ratanatharathorn A, Koenen KC, Chibnik LB, Weisskopf MG, Rich-Edwards JW, Roberts AL. Polygenic risk for autism, attention-deficit hyperactivity disorder, schizophrenia, major depressive disorder, and neuroticism is associated with the experience of childhood abuse. *Mol Psychiatry*. 2021;26(5):1696-1705. doi:10.1038/s41380-020-00996-w

69. Strom NI, Smit DJA, Silzer T, et al. Meta-analysis of genome-wide association studies of hoarding symptoms in 27,651 individuals. *Transl Psychiatry*. 2022;12(1):1-8. doi:10.1038/s41398-022-02248-7

70. Zhang R, Birgegård A, Fundín B, et al. Association of autism diagnosis and polygenic scores with eating disorder severity. *Eur Eat Disord Rev J Eat Disord Assoc*. 2022;30(5):442-458. doi:10.1002/erv.2941

71. Nayar K, Sealock JM, Maltman N, et al. Elevated Polygenic Burden for Autism Spectrum Disorder Is Associated With the Broad Autism Phenotype in Mothers of Individuals With Autism Spectrum Disorder. *Biol Psychiatry*. 2021;89(5):476-485. doi:10.1016/J.BIOPSYCH.2020.08.029

72. Ding Y, Hou K, Xu Z, et al. Polygenic scoring accuracy varies across the genetic ancestry continuum. *Nature*. Published online May 17, 2023:1-8. doi:10.1038/s41586-023-06079-4
