# Supplementary material for: Systematic Review and Meta-Analysis: Phenotypic Correlates of the Autism Polygenic Score
Source: JAACAP Open. 2025 Apr 14;3(4):839–51. doi: 10.1016/j.jaacop.2025.04.001 (PMC12684455; doi:10.1016/j.jaacop.2025.04.001)
Supplement: Supplement 3 [file mmc3.docx]

See Supplement 3, available online: <https://doi.org/10.6084/m9.figshare.29136083>
